# Physiological Reference Mapping Reveals Context-Dependent Dysregulation of Regenerative Programs in Duchenne Muscular Dystrophy

**DOI:** 10.3390/cells15191743

**Published:** 2026-09-24

**Authors:** Laura Perin, Yan-Yun Liu, Gregory A. Brent, Anna Milanesi

**Affiliations:** 1 GOFARR Laboratory, Children’s Hospital Los Angeles, Division of Urology, Saban Research Institute, Los Angeles, CA 90027, USA; lperin@chla.usc.edu; 2 Keck School of Medicine, University of Southern California, Los Angeles, CA 90027, USA; 3 Department of Medicine, Division of Endocrinology, Diabetes and Metabolism, David Geffen School of Medicine at UCLA—VA Greater Los Angeles Healthcare System, 11301 Wilshire Boulevard, Los Angeles, CA 90027, USAgbrent@mednet.ucla.edu (G.A.B.)

**Keywords:** Duchenne muscular dystrophy, muscle stem cells, physiological reference mapping

## Abstract

**Highlights:**

**What are the main findings?**

- Comparison with normal regeneration reveals distinct abnormalities in dystrophic muscle: excessive extracellular matrix (ECM) transcription and an attenuated MYC response in activated muscle stem cells.
- Spatial analysis shows that dystrophic transcriptional changes reflect both altered tissue composition and changes within comparable muscle niches.

**What are the implications of the main findings?**

- The biological reference matters: comparison with resting muscle alone can obscure the distinction between normal repair and disease-associated changes.
- Mapping dystrophic muscle against normal regenerative states provides a framework for understanding how repair becomes dysregulated in Duchenne muscular dystrophy.

**Abstract:**

Duchenne muscular dystrophy (DMD) involves chronic degeneration and regeneration, causing dystrophic muscle stem cells (MuSCs) to differ from uninjured wild-type tissue. Conventional disease-versus-resting-control comparisons may therefore conflate dysregulation with regeneration. We developed a physiological reference-mapping framework integrating normal regeneration trajectories with replicated dystrophic datasets to estimate disease-associated deviations relative to regenerative state and spatial context. We applied it across single-cell/single-nucleus, bulk, and spatial RNA-sequencing datasets. In a replicated genotype-by-state MuSC dataset comprising wild-type (WT) and dystrophin-deficient mdx mouse samples across four lineage states, with three biological replicates per group, activated mdx MuSCs showed excessive extracellular matrix induction (standardized mdx-WT difference = 1.431; FDR = 3.69 × 10^−9^) and an attenuated MYC-associated response (−0.731; FDR = 2.57 × 10^−4^) among five prespecified programs. Spatial decomposition indicated substantial within-niche remodeling beyond altered niche composition. Physiological calibration identified extracellular matrix induction as a normal transient program that becomes excessive in dystrophy and suggested that reduced oxidative phosphorylation transcription relative to resting muscle may represent insufficient suppression relative to normal regeneration. An independent human dystrophinopathy spatial cohort supported increased whole-biopsy extracellular matrix transcription. Physiological reference mapping therefore helps distinguish regenerative adaptation from context-dependent disease dysregulation in DMD.

## 1. Introduction

Duchenne muscular dystrophy (DMD) is caused by loss of functional dystrophin and is characterized by repeated myofiber degeneration, inflammation, fibrosis, and incomplete regeneration [1]. Muscle stem cells (MuSCs) are essential for skeletal muscle repair, and their transition from quiescence through activation, proliferation, and differentiation is tightly coordinated with changes in the surrounding niche [2,3,4]. Dystrophin also has a cell-intrinsic role in MuSC polarity and asymmetric division, providing a direct mechanistic link between the DMD mutation and altered stem cell behavior [5]. Nevertheless, the transcriptome measured in dystrophic muscle reflects more than the primary genetic lesion: it also captures chronic regenerative activity, shifts in cell composition, and remodeling of the tissue microenvironment [6]. Normal muscle regeneration is not a single comparator state. Single-cell studies of acute injury have resolved a temporally ordered and heterogeneous response in which MuSCs and their descendants move through quiescent, activated, proliferative, and differentiating states while immune, stromal, vascular, and myofiber compartments change in parallel [7,8]. Each stage has an expected molecular profile. A pathway that is low relative to homeostatic/quiescent wild-type muscle may therefore be appropriate for an activated regenerative state, whereas a pathway that is increased during normal repair may become pathological only when its magnitude, timing, persistence, or localization exceeds the physiological range [6,9,10].

This creates a central interpretive problem for transcriptomic studies of DMD. Conventional DMD-versus-resting-WT contrasts can conflate disease-associated dysregulation with displacement along the normal regenerative trajectory. The same concern applies to spatial data, where differences between whole-tissue sections may result from altered niche abundance, altered expression within a matched niche, or both. Treating individual cells or spatial spots as independent biological replicates can further overstate evidence. Consequently, identifying a molecular difference is not sufficient to establish that the difference represents a disease-specific deviation.

Healthy reference mapping provides an important precedent for separating cell state composition from within-state molecular alteration. Dann and colleagues showed that disease-associated single-cell states can be identified more precisely by comparison with healthy reference atlases, explicitly distinguishing changes in state abundance from transcriptional alteration within a state [11]. Methods for multi-condition trajectory analysis similarly permit formal comparison of lineage structure and gene expression dynamics across conditions [12,13]. These approaches establish the value of reference-based analysis.

Evidence from other regenerative organs reinforces the need for this distinction. Transitional programs that are adaptive during acute repair can become persistent or maladaptive in chronic disease, as illustrated by KRT8-positive transitional epithelial states in pulmonary fibrosis and by divergent adaptive and fibrotic trajectories during kidney repair [14,15]. Disease biology may therefore involve an excessive, attenuated, delayed, persistent, or spatially redirected version of a normal physiological response rather than an entirely novel program. This logic is especially relevant to DMD, in which degeneration and regeneration recur asynchronously throughout the tissue.

Here, we developed a physiological reference framework for interpreting transcriptional dysregulation in dystrophic muscle. Conceptually, the observed dystrophic program is decomposed into the expected normal program at the matched regenerative state, time, and niche plus a context-dependent disease-associated deviation. We applied this framework across independent mouse single-cell and single-nucleus datasets, a replicated genotype-by-state MuSC experiment, normal injury time courses, spatial transcriptomic sections, and a human dystrophinopathy cohort. Biological inference was performed at the animal, library, or donor level, as appropriate, rather than at the cell or spot level.

We asked whether physiological matching could distinguish three biologically different outcomes: a response compatible with normal regeneration, a normal response that becomes excessive or insufficient in dystrophy, and a difference that remains unresolved because the available reference or replication is inadequate. We further tested whether tissue-level abnormalities could be explained by altered niche composition or persisted within matched niches, and whether the strongest mouse residual generalized to human dystrophinopathy. The objective was not to nominate a single pathway as the defining mechanism of DMD, but to establish a reproducible analytical strategy for determining which transcriptomic abnormalities remain after normal regenerative state and spatial context are taken into account.

## 2. Methods

### 2.1. Study Design and Analytical Principles

The analysis was organized as a sequence of discovery, eligibility, freezing, and validation stages. Datasets could contribute to state mapping without being eligible for genotype inference. Eligibility required identifiable biological replicates, compatible states or niches, adequate signal, and a prespecified statistical unit. Cells, nuclei, spots, and computational clusters were never treated as independent animals or donors. Pathway definitions and decision rules were frozen before confirmatory cross-dataset tests whenever possible. Comparing disease profiles with matched healthy reference states can improve the identification of disease-associated cellular phenotypes [11]. We extended this principle by using the normal regenerative trajectory as a physiological reference and represented the framework conceptually as

*Y*_DMD_(c) = *µ*_normal_(c) + *D*_DMD_(c),

where *Y*_DMD_(c) denotes the observed molecular state in dystrophic muscle, *µ*_normal_(c) denotes the expected physiological state during normal regeneration, and *D*_DMD_(c) denotes the disease-associated deviation conditional on biological context (c). Context included cell state, regenerative time, spatial niche, tissue and genetic background. The equation was used as a conceptual representation; disease effects were estimated within individual datasets rather than by direct subtraction of measurements across platforms.

### 2.2. Datasets

The datasets, biological replication, experimental systems, and analytical roles are summarized in Table 1, with detailed accession-specific processing described in Supplementary Materials Methods S1–S21.

### 2.3. Single-Cell and Single-Nucleus Processing

PRJNA771932 [16] matrices were imported separately by mouse. Nuclei were retained with at least 300 detected genes, mitochondrial RNA no greater than 20%, and log10 genes per unique molecular identifier (UMI) of at least 0.80. Doublets were identified independently within each mouse. The atlas was analyzed without batch integration to avoid removing genuine disease structure. Counts were log-normalized, 3000 highly variable genes were selected, mitochondrial percentage was regressed during scaling, and graph clustering was evaluated across multiple resolutions. Canonical markers were used for broad annotation. MuSC and myogenic clusters were then reprocessed and classified as homeostatic/quiescent, dystrophic PAX7-positive, activated, cycling, or differentiating states.

For pseudobulk inference, raw counts were summed within each biological mouse or donor and the relevant state. A state profile required a prespecified minimum number of cells or nuclei. edgeR quasi-likelihood negative-binomial models were fitted to biological replicate pseudobulks. Nucleus-level models and pseudotime associations were treated as descriptive unless independently aggregated by animal.

### 2.4. Normal Physiological References and State Mapping

GSE163856 [17] provided a normal acute activation reference from PAX7-positive MuSCs. The replicated bulk series was sampled 0, 15, 30, 60, 90, and 120 min following MuSC isolation and early activation; a complementary fixed Smart-seq2 experiment was performed at T0, T2, and T4 hours. Time-associated signatures were derived using normal samples only. Cross-platform mapping used within-cell or within-sample ranks rather than direct comparison of raw expression magnitudes. Closest-state assignments were interpreted as relative similarity, not definitive labels. Independent spatial injury references were used to evaluate whether pathway direction was reproducible during normal regeneration. Scores were interpreted within each dataset. Raw pathway scores were not pooled across studies, platforms, muscles, or injury models.

### 2.5. Frozen Pathway Definitions and Matched Residual Models

We analyzed five predefined gene programs: a collagen-associated extracellular matrix (ECM) program comprising Col1a1, Col1a2, Col3a1, Col5a1 and Col5a2, and four Hallmark gene sets representing oxidative phosphorylation (OXPHOS), MYC Targets V1, mTORC1 signaling and TNF/NF-κB signaling. Gene set membership was kept constant throughout the statistical analyses, with genes matched to their mouse counterparts where required. The ECM score summarizes expression of the five collagen genes, while the OXPHOS score summarizes expression of genes involved in oxidative phosphorylation; neither directly measures deposited collagen or mitochondrial function. For GSE268313 [9], the primary model was pathway score ~ genotype × state. WT and mdx each contributed three biological replicates in freshly isolated MuSCs, 3-day post-injury activated MuSCs, cultured myoblasts, and day 2 myotubes. No pairing across states was assumed. Libraries were analyzed as independent biological replicates. Gene-level analyses used trimmed mean of M-values (TMM) normalization, robust dispersion estimation and quasi-likelihood testing. Hallmark programs were assessed with the correlation-adjusted mean rank gene set test (CAMERA) to account for inter-gene correlation. Program score contrasts used t-based 95% confidence intervals with 16 residual degrees of freedom and Benjamini–Hochberg (BH) correction across five programs within each state. Standardized score differences are not Cohen d effect sizes.

For GSE225766 [18], frozen cluster-level pathway summaries were collapsed to one observation per mouse, validated niche, and pathway using spot-number-weighted means of cluster medians. The primary model was pathway score ~ genotype × validated niche with a random intercept for mouse. Contractile, ECM/ fibro-adipogenic progenitor (FAP), and perivascular niches were defined without using pathway outcomes. The final spatial input contained 115 unique mouse–niche–pathway observations from three WT and five mdx mice. These are repeated niche/program records, not 115 independent animals. Contractile and ECM/FAP comparisons included three WT and five mdx mice; perivascular comparisons included three WT and four mdx mice. Restricted maximum-likelihood models used a mouse intercept, with the retained conservative shared denominator degrees of freedom and BH correction across five programs per niche. We calculated the percentage contribution of tissue composition and within-niche expression changes from their sizes, irrespective of direction.

### 2.6. Robustness, Coherence, and Decision Gates

MuSC models were refitted after leaving out each of the 24 libraries and with heteroscedasticity-consistent (HC3) errors. Spatial models were refitted after leaving out each mouse and under weighted and unweighted aggregation. Pathway findings were decomposed to member genes; coherence required distributed directional support rather than dominance by one gene. Spatial projection was repeated under alternative accepted niche mappings. Exact donor-label permutations were used for human spatial tests, followed by BH correction across frozen programs or niche–program tests. Outcomes were classified as confirmed unchanged, confirmed but refined, physiologically recontextualized, directionally reinterpreted, spatially or compositionally conditional, or unresolved. A result was unresolved when the necessary normal state, biological replication, tissue match, or measurement resolution was absent. BH-adjusted *p* values are denoted as q or false discovery rate (FDR).

### 2.7. Structured Extraction and Physiological Reinterpretation of Published Claims

Published claims from seven purposively selected dystrophinopathy studies were standardized and frozen before physiological matching. Claim-specific eligibility required a compatible state-, time-, tissue-, or niche-matched reference and adequate biological replication and measurement resolution. Eligible claims were assigned one prespecified interpretive outcome; ineligible claims remained unresolved. Disease effects were estimated within source datasets, with external references supplying the expected physiological context rather than cross-platform subtraction. Full operational procedures, record fields, eligibility rules, outcome definitions, denominators, and row-level justifications are provided in Supplementary Materials Method S11 and Supplementary Table S1. The workflow is illustrated in Supplementary Figure S1.

Biological replication was defined from source-study animal, donor or library identifiers. Cells, nuclei and spots were not counted as independent biological replicates. For human whole-biopsy scores, raw in-tissue counts were summed first, normalized by all-gene count totals, log2(CPM + 0.5)-transformed and gene-standardized before averaging fixed program genes. Counts per million (CPM) therefore use all genes as the denominator. HC1 and HC3 represented one donor and their biopsy scores were averaged, yielding three control and eight disease donors. Regional scores averaged eligible spot scores before donor collapse. Exact tests enumerated 165 assignments of three control labels among 11 donors; BH families comprised five programs, five collagen genes or ten region–program comparisons. Approximate Welch 95% intervals were reported separately from permutation *p* values. Donor correction does not remove age or muscle confounding or guarantee exchangeability. Analyses without sufficient replication or reference correspondence remained descriptive. Full procedures and a panel-level statistical ledger are provided in the Supplementary Materials.

### 2.8. Additional Computational Analyses

We extended the analyses to examine cellular sources of ECM transcription, regeneration-associated pathways and responses to dystrophin restoration. Collagen contributions and cell type pseudobulk scores were quantified per mouse, with marker exclusion analyses assessing the sensitivity of myogenic findings. In eight human dystrophinopathy donors, regression and exact Spearman tests assessed the relationship between ECM scores and connective tissue representation. Four Hallmark gene sets, MYOGENESIS, NOTCH_SIGNALING, E2F_TARGETS and G2M_CHECKPOINT, were evaluated using program score models and CAMERA, with separate BH corrections for WT transitions, genotype contrasts and interactions (Supplementary Materials Methods S15–S17; Figure S2–S4).

Foxo1/Foxo3 expression was analyzed using edgeR, and transcription factor activity was inferred from CollecTRI targets using univariate linear models [24]. Immune–satellite cell communication was assessed per mouse using LIANA with CellPhoneDB scores [25]. Treatment-associated changes were examined in four exon-23-targeting antisense oligonucleotide (e23AON)-treated mice from PRJNA771932 using mouse-level pseudobulk scores (Supplementary Materials Methods S18–S20; Figure S5–S8).

The same five programs were also evaluated in bulk quadriceps RNA-sequencing data from the independent AAV9-CRISPR/Cas9 study GSE180771 [23]. Four-week and eighteen-month sedentary cohorts were analyzed separately using TMM normalization, program score models, CAMERA and exact permutation tests. Treatment comparisons were corrected for multiple testing within each predefined family; the exercise cohort was analyzed descriptively because sequencing platform and experimental group were confounded (Supplementary Materials Method S21; Figure S9).

### 2.9. Software and Reproducibility

Analyses were performed in R version 4.6.1 using Seurat v5.5.1, scDblFinder v1.26.7, edgeR v4.10.3, limma v3.68.5, decoupleR v2.17.0, msigdbr v26.1.0, mgcv v1.9-4, and ggplot2 v4.0.3. CAMERA gene set testing was performed using limma. CollecTRI regulatory interactions were accessed as a network resource through the decoupleR/OmniPath ecosystem and were not treated as a separate R package. Additional analyses used Python 3.12.14, LIANA v1.10.0, scikit-learn v1.9.1, pandas v2.3.3 and SciPy v1.18.1. Source tables are provided in the supplementary source-data archive. Analysis scripts, software version records and resource snapshots are available from the corresponding author upon reasonable request.

## 3. Results

**Dystrophic MuSCs are displaced from the resting state along a physiological activation axis.** We first asked whether mdx MuSCs occupy transcriptional states comparable to homeostatic/quiescent wild-type (WT) MuSCs or are displaced along a normal regenerative activation axis (Figure 1A).

Analysis of the MuSC/myogenic compartment of PRJNA771932 identified homeostatic quiescent MuSCs, dystrophic Pax7-positive MuSCs, Runx1-positive-activated progenitors, cycling myogenic progenitors, and differentiating myogenic cells (Figure 1B). Visualization by experimental condition showed substantial genotype-associated segregation across this state space (Figure 1C). Importantly, this difference was also evident at the level of biological replicates rather than only at the level of individual nuclei. The three WT mice were dominated by homeostatic quiescent MuSCs, whereas the four mdx mice contained variable contributions from activated, cycling, and differentiating myogenic states (Figure 1D).

To determine whether this displacement corresponded to physiological MuSC activation, we next compared each biological replicate with an independent normal activation reference derived from GSE163856. The reference comprised transcriptional signatures representing quiescent (T0), early-activation (T2), and activation/commitment (T4) states (Figure 1E). WT samples showed the greatest relative similarity to the quiescent T0 reference, whereas several mdx samples showed greater similarity to the T2 or T4 activation references (Figure 1F). These projections represent relative transcriptional similarity and were not interpreted as exact elapsed-time or pseudotime assignments. Together, these observations demonstrate that dystrophic and homeostatic/quiescent WT MuSC populations differ substantially in regenerative state composition. Consequently, a conventional mdx-versus-WT comparison can combine genotype-associated transcriptional differences with displacement along a physiological MuSC activation axis, providing the rationale for estimating disease-associated deviations relative to regenerative state.

**State-matched analysis reveals excessive ECM induction and attenuated MYC-associated transcriptional response in dystrophic MuSCs.** We next asked which transcriptional abnormalities remain when WT and mdx cells are compared within corresponding MuSC-lineage states. We analyzed GSE268313, a replicated bulk RNA-sequencing dataset comprising WT and mdx cells across four lineage states: freshly isolated satellite cells (FiSCs), activated satellite cells collected 3 days after injury (ASC 3 dpi), myoblasts, and myotubes, with three biological replicates per genotype–state combination. Principal component analysis (PCA) of the 24 libraries showed strong separation according to lineage state, with PC1 and PC2 accounting for 52.2% and 25.9% of total transcriptomic variation, respectively (Figure 2A). Thus, cell state represented the dominant source of transcriptomic variation in this dataset. We evaluated five prespecified molecular programs representing ECM, OXPHOS, MYC targets, mTORC1 signaling, and TNF/NF-κB signaling. Their behavior varied substantially across lineage progression and between genotypes, indicating that disease-associated transcriptional effects were state-dependent rather than uniform across the MuSC lineage (Figure 2B). The strongest positive disease-associated deviation occurred in the ECM program during MuSC activation.

Although ECM program scores changed during lineage progression in both genotypes, activated mdx MuSCs showed markedly greater ECM induction than state-matched WT cells (standardized mdx-WT difference = 1.431, 95% CI 1.195–1.667, FDR = 3.69 × 10^−9^; Figure 2C). MYC showed a qualitatively different abnormality: MYC-associated transcription increased during WT MuSC activation, whereas this response was attenuated in activated mdx MuSCs (standardized mdx-WT difference = −0.731, 95% CI −1.034 to −0.428, FDR = 2.57 × 10^−4^; Figure 2D). Analysis of state-specific genotype effects across all five prespecified programs demonstrated that dystrophic transcriptional dysregulation could not be represented by a uniform direction or pathway behavior (Figure 2E). Although the design was balanced, estimates were based on three biological replicates per genotype–state group; therefore, leave-one-library-out analyses were used to assess dependence on individual samples. The activated-state ECM and MYC effects retained their direction and standard-model BH significance in all 24 leave-one-library-out analyses; HC3 significance persisted in 24/24 ECM and 22/24 MYC refits (Figure 2F). Together, these results identify excessive ECM induction and attenuated MYC-associated transcriptional response as robust but directionally distinct abnormalities during dystrophic MuSC activation.

**Disease-associated transcriptional remodeling persists within matched dystrophic muscle niches.** Dystrophic muscle undergoes extensive remodeling of cellular and tissue composition. We therefore asked whether disease-associated transcriptional differences were primarily attributable to altered niche abundance or whether transcriptional abnormalities persisted within corresponding tissue environments. Spatial transcriptomic analysis of GSE225766 identified contractile-, ECM/FAP-, perivascular-, regenerating-, and immune-enriched niches (Figure 3A). Regenerating- and immune-enriched regions were reproducibly detected in dystrophic tissue but lacked corresponding evaluable WT regions and were therefore retained descriptively rather than included in matched-niche genotype inference. Analysis at the level of individual biological replicates demonstrated pronounced remodeling of dystrophic tissue architecture, including expansion of regenerating- and immune-enriched regions and a reduction in the relative contractile compartment (Figure 3B).

To distinguish compositional remodeling from transcriptional changes within common niches, we decomposed the tissue-level disease contrast into contributions associated with altered niche abundance and within-niche transcriptional differences. For each of the five prespecified programs, a substantial proportion of the observed contrast was attributable to within-niche remodeling: 84% of the total contribution for ECM, 87% for mTORC1, 94% for MYC, 86% for TNF/NF-κB, and 62% for OXPHOS (Figure 3C). OXPHOS showed a more balanced decomposition, with 62% of the total contribution assigned to the within-niche component, indicating a comparatively greater contribution from altered niche composition than observed for the other programs. These values represent decomposition of the observed tissue-level contrast and were not interpreted as proportions of variance explained. Matched-niche analysis confirmed significant genotype-associated effects across multiple transcriptional programs (Figure 3D). ECM showed the most consistent spatial abnormality, with significantly higher ECM program scores in D2-mdx than WT muscle within the contractile niche (difference = 0.938, FDR = 0.00105), ECM/FAP niche (difference = 0.803, FDR = 0.00273), and perivascular niche (difference = 1.182, FDR = 0.000389; Figure 3E). Inference was performed using mouse × niche summaries rather than individual spatial spots as biological replicates. The ECM effect was also insensitive to individual animals. Leave-one-mouse-out analyses retained a positive and statistically significant D2-mdx − WT ECM difference within each of the three matched niches in every iteration (Figure 3F). Thus, altered tissue composition contributes to the dystrophic transcriptional phenotype but does not fully account for it; substantial disease-associated transcriptional remodeling persists within matched tissue niches.

**Physiological injury trajectories distinguish regenerative responses from disease-associated deviations.** We next asked whether transcriptional programs conventionally classified as abnormal in dystrophic muscle might also represent components of the normal regenerative response. To address this question, we positioned disease-associated pathway states relative to independent normal-injury references. ECM program scores increased progressively in the regeneration-enriched spatial niche between days 1 and 5 after normal muscle injury in GSE225766 (Figure 4A). Because only one section was available at each injury time point, this trajectory was considered descriptive rather than inferential. Nevertheless, it demonstrated that ECM induction itself is a feature of normal early regeneration. D2-mdx denotes dystrophin-deficient mdx mice on the DBA/2J background. This cohort was used because GSE225766 provides the replicated spatial disease data together with normal injury landmarks; it was not chosen after ranking disease effects. When the five D2-mdx mice were positioned relative to this normal-injury reference, their ECM scores exceeded the range observed across the sampled normal-injury interval (Figure 4B). Gene-level analysis in the same dataset provided complementary evidence for coordinated ECM dysregulation. All five genes in the prespecified collagen program, *Col1a1*, *Col1a2*, *Col3a1*, *Col5a1*, and *Col5a2*, were increased in D2-mdx tissue, with each of the five dystrophic mice exceeding the WT median for every collagen gene. The collagen gene family remained significant after multiple-testing correction (FDR = 0.037; Figure 4C). These observations indicate that ECM induction is not intrinsically disease-specific; rather, its magnitude becomes abnormal relative to the physiological regenerative response.

OXPHOS provided a contrasting example of physiological recontextualization. In normal injured muscle, OXPHOS remained suppressed during days 1–3 and increased by day 5, consistent with early transcriptional suppression followed by recovery (Figure 4D). A similar recovery pattern was observed across three independent normal-injury spatial datasets: later-versus-earlier comparisons of measurable OXPHOS genes showed a positive central tendency in GSE225766 (day 5 versus day 1), GSE161318 (day 7 versus day 2), and GSE223813 (day 8 versus day 4; Figure 4E). This trajectory changed the interpretation of the static disease-versus-control contrast. Although reduced OXPHOS in dystrophic relative to WT muscle might conventionally be interpreted as impaired oxidative metabolism, comparison with the normally suppressed regenerative state suggested a different possibility. Relative to the sparsely sampled normal regenerative trajectory, the spatial dystrophic OXPHOS pattern was compatible with insufficient transcriptional suppression relative to the sampled reference (Figure 4F). This represents a framework-generated hypothesis rather than a definitive mechanistic conclusion and does not establish a replicated MuSC-autonomous OXPHOS defect.

**Human whole-biopsy ECM association is retained after donor correction.** We first tested the frozen mouse early-activation attenuation module in human MuSC nuclei from GSE288958. The prespecified tissue-matched comparison did not reproduce the mouse signal. An apparent supportive pattern emerged only when anatomically unmatched control samples were included and was therefore classified as tissue-confounded. Because the dataset lacked an adequately replicated activated control MuSC population, it could not provide a definitive test of the activated-state residual (Supplementary Materials Method S10). In the independent human spatial cohort, four control biopsies represented three donors and eight dystrophinopathy biopsies represented eight donors. Whole-biopsy pseudobulk scores were calculated using all-gene count normalization. Scores from the two biopsies contributed by the same control donor (HC1 and HC3) were averaged, so that donors constituted the statistical units of analysis (Figure 5A). Among the five prespecified programs, ECM was the only signal that passed donor-level BH correction, with significantly higher ECM program scores in dystrophinopathy than controls (difference = 1.718; approximate Welch 95% CI 0.982–2.453; exact *p* = 0.00606; BH q = 0.0303; Figure 5B). MYC, mTORC1, TNF/NF-κB, and OXPHOS were not significant after correction. The ECM finding was supported at the gene level. All five prespecified collagen genes, *COL1A1*, *COL1A2*, *COL3A1*, *COL5A1*, and *COL5A2*, were elevated in dystrophinopathy biopsies and passed correction within the collagen gene family (q = 0.00758–0.0121; Figure 5C). ECM scores remained higher in dystrophinopathy across donor exclusion analyses, although statistical significance was sensitive to the small control group (Supplementary Materials Method S10).

We further examined whether the ECM abnormality localized to tissue regions associated with regenerative biology. ECM program scores were higher in dystrophinopathy within both MuSC-enriched regions (difference = 0.828) and regeneration-enriched regions (difference = 0.938), although neither comparison reached significance after multiple-testing correction (both q = 0.0848; Figure 5D). Because Visium-defined regions contain mixtures of cell types, these findings do not establish MuSC-specific ECM production. Together, the human data support increased whole-biopsy ECM transcription, consistent with the strongest disease-associated program identified in mice, while regional differences remain unresolved. Because the human cohort was cross-sectional and lacked a corresponding normal-injury time course, it could not test the temporal component of physiological reference mapping.

To identify the cellular contributors to collagen transcription, we examined the full mouse single-nucleus atlas, comprising 22,417 nuclei from three WT and four mdx mice. Stromal populations accounted for 88.3–92.5% of captured collagen transcripts in mdx muscle (Supplementary Figure S2). Collagen transcripts remained detectable in the broader myogenic compartment after marker exclusion, but could not be attributed conclusively to MuSCs. We also examined whether human ECM scores increased with connective tissue representation. Across eight dystrophinopathy donors, the association was positive but not statistically significant (Spearman ρ = 0.524; *p* = 0.197; Supplementary Figure S3).

Additional regeneration-associated programs recovered expected transitions in WT cells: E2F and G2M scores increased during activation and declined during differentiation, while myogenesis increased from myoblasts to myotubes. These patterns were supported by both program score models and CAMERA (Supplementary Figure S4). Activated mdx samples showed higher myogenesis and lower E2F/G2M scores, also supported by CAMERA, whereas NOTCH findings differed between methods.

Activated mdx samples also showed increased Foxo1 and Foxo3 expression and higher inferred target–program scores (Supplementary Figures S5 and S6). Excluding shared targets retained support for FOXO1 but weakened the FOXO3 result, indicating that the two inferred responses were not equally robust. Analysis of 38 eligible immune–satellite interaction pairs identified no genotype differences after multiple-testing correction (Supplementary Figure S7).

Finally, we examined whether dystrophin restoration was accompanied by changes in the five transcriptional programs. In PRJNA771932, e23AON-treated mice showed lower stromal ECM scores than untreated mdx mice, although treatment differences did not reach significance after multiple-testing correction (Supplementary Figure S8). In contrast, AAV9-CRISPR/Cas9-mediated dystrophin correction in ΔEx44 mice from GSE180771 [23] was associated with significant transcriptional changes: increased OXPHOS at 4 weeks (q = 0.0208), reduced TNF/NF-κB at 4 weeks and 18 months (q = 0.0208 and 0.00533), and reduced ECM at 18 months (q = 0.00533). These changes were opposite in direction to the disease-associated differences and were corroborated by CAMERA (Supplementary Figure S9). Together, these results support partial reversal of disease-associated transcriptional changes following dystrophin correction.

**Physiological reference matching refines the interpretation of published dystrophic muscle findings.** Finally, we asked whether physiological recontextualization extended beyond the prespecified pathway examples used to develop the framework. We performed a structured claim-level audit of 79 claim records from seven dystrophic muscle publications. Published claims were standardized and frozen before physiological reinterpretation and were evaluated only when an eligible state-, time-, tissue-, or niche-matched physiological reference was available (Supplementary Figure S10; Supplementary Table S1).

Of the 79 frozen claims, 48 had eligible physiological references and could be classified. Nineteen (40%) were confirmed but required refinement of their biological interpretation, sixteen (33%) were spatially or compositionally conditional, nine (19%) were confirmed without substantive reinterpretation, and four (8%) were physiologically recontextualized. The remaining 31 claims were retained as unresolved because the available evidence lacked an appropriate physiological reference, sufficient biological replication, matched activation state, genotype background resolution, or other required correspondence (Supplementary Figure S10A,C). Importantly, physiological matching was not designed to classify previously published observations as correct or incorrect. Instead, it assessed whether their biological interpretation changed after accounting for the physiological state represented by the disease tissue. Most evaluable claims were therefore not negated by physiological matching; rather, they were refined or became conditional on regenerative state, tissue composition, or spatial context. Retaining an unresolved category further prevented technically ineligible observations from being forced into positive or negative conclusions.

## 4. Discussion

Applying physiological reference matching showed that several apparent dystrophy-associated changes reflected displacement along normal regenerative programs, whereas others persisted as state- or niche-matched disease-associated deviations. DMD muscle undergoes chronic and spatially heterogeneous degeneration, inflammation and regeneration, producing cell states that may have no direct counterpart in uninjured tissue [16,18]. Consequently, a conventional disease-versus-resting-control comparison combines at least two components: displacement along a normal regenerative program and a disease-associated deviation from that program. By incorporating normal regenerative trajectories and matching dystrophic samples by cell state, injury interval and spatial niche, the present framework separates these components and produces more specific biological interpretations. This distinction was evident in the MuSC compartment. Dystrophic Pax7-positive cells were displaced from the homeostatic quiescent state toward activated and differentiating states, consistent with the chronic regenerative environment of dystrophic muscle. Normal-injury studies demonstrate that MuSC activation rapidly reorganizes transcription before overt proliferation and differentiation [17,19]. Therefore, differences between dystrophic MuSCs and resting wild-type MuSCs cannot automatically be classified as disease-specific defects. After matching genotype within four MuSC-lineage states in a replicated dataset, however, substantial residual abnormalities remained, including excessive ECM program scores and an attenuated MYC-associated transcriptional response in activated MuSCs [9]. Thus, physiological matching preserves disease biology while separating activation-compatible changes from persistent deviations.

ECM provides the clearest example of a physiological program that becomes quantitatively and spatially abnormal in dystrophy. Matrix remodeling is an essential component of normal muscle repair, supported by the coordinated activity of FAPs, connective tissue fibroblasts and myogenic cells [26,27]. The presence of an ECM signature in dystrophic muscle is therefore not, by itself, evidence of a disease-specific process. In the present analyses, the abnormality was defined more precisely by excess magnitude and spatial context relative to the normal regenerative reference. ECM program scores were elevated in activated mdx MuSCs, across matched spatial niches in D2-mdx muscle and in human dystrophinopathy biopsies. Five collagen genes also showed concordant directional effects, supporting the interpretation of coordinated matrix remodeling rather than a score driven by a single transcript. The independent gene-editing extension provides evidence that several tissue-level transcriptional programs respond to dystrophin restoration. Lower ECM transcription at 18 months and lower TNF/NF-κB were accompanied by increased OXPHOS transcription at 4 weeks. The human findings extend the ECM observation from mouse models to dystrophinopathy biopsies, supporting its relevance across species. This support is strongest at the whole-biopsy level; regional differences and the other transcriptional programs were not statistically supported. The small control cohort and differences in age and sampled muscle warrant confirmation in larger, better-matched cohorts.

Spatial matching further demonstrated that these effects could not be explained solely by altered tissue composition. Dystrophic muscle showed extensive changes in the abundance of contractile, regenerating, ECM/FAP, immune and perivascular niches, as expected from its pathological architecture [18]. The within-niche component accounted for 84% of the absolute ECM decomposition contrast. ECM was elevated within contractile, ECM/FAP and perivascular regions, and these effects remained directionally stable after omission of individual mice. Thus, tissue remodeling contributes substantially to the dystrophic transcriptome, but it does not fully account for the observed molecular abnormalities.

MYC and OXPHOS illustrated distinct outcomes of physiological calibration. In the replicated GSE268313 MuSC-lineage series, the MYC response was specifically attenuated in activated mdx MuSCs (Figure 2D–F), consistent with incomplete activation of the wild-type MYC program rather than a uniform lineage-wide reduction. Across normal-injury spatial references, OXPHOS showed early suppression followed by recovery (Figure 4D,E), consistent with stage-dependent MuSC metabolic remodeling: activation shifts cells away from oxidative metabolism toward glycolysis, whereas mitochondrial respiration and oxidative phosphorylation increase during commitment and differentiation [28,29]. Thus, a static finding of lower OXPHOS in dystrophic versus resting wild-type muscle may be misleading. Relative to the matched regenerative expectation, the dystrophic state was compatible with insufficient OXPHOS transcriptional suppression relative to the sampled reference.

FOXO factors add another state-dependent context. Previous studies have linked FOXO3 to Notch-dependent quiescence and self-renewal [30], and PTEN-dependent regulation of FOXO1 localization to the maintenance of MuSC quiescence [31]. Their increased expression in activated mdx cells may therefore reflect altered coordination between activation and preservation of the stem cell pool. Whether this represents a compensatory response or contributes to impaired regeneration remains unresolved.

Together, these findings support a unified physiological reference-mapping model in which apparent disease-associated differences may represent regenerative state displacement, excessive or persistent physiological responses, attenuated responses, directionally reinterpreted responses, or context-dependent tissue remodeling (Figure 6). Applying the framework to claims from seven published studies showed how regenerative state and tissue composition can qualify the interpretation of reported disease-associated changes. This extends its use beyond individual pathway analyses, although it does not constitute independent validation.

Several limitations should be considered. The public datasets provide complementary views of DMD but include small cohorts with differences in age, muscle and experimental design. Although sensitivity analyses supported the main mouse findings, larger independent cohorts are needed to assess their generalizability. The MuSC experiment contained three libraries per group, and shared animal or culture origins across states could not be established. Normal-injury references were sparsely sampled, while the human data were cross-sectional and affected by age and muscle differences, limiting conclusions about regenerative timing. Cell and niche annotations may also encompass mixed populations and technical uncertainty; stromal labels partly relied on collagen markers. Communication and treatment analyses examined broad cell populations, and treatment annotations were checked against the existing atlas without independent validation. Finally, the findings describe transcriptional changes; functional studies are needed to establish their consequences for matrix deposition, protein activity and muscle repair.

In conclusion, normal regenerative references refine the interpretation of transcriptional changes in dystrophic muscle. The framework distinguishes physiological state displacement from disease-associated deviation, separates compositional remodeling from within-niche dysregulation and preserves unresolved or negative results. Applied to DMD, it identifies excessive ECM remodeling relative to the sampled normal regenerative trajectory as the most reproducible cross-platform and cross-species residual, while showing that other apparent abnormalities depend on cell state, spatial context, or the selected physiological comparator.

### Data and Code Availability

All datasets analyzed in this study are publicly available from the repositories and accession numbers listed in Table 1. Figure and table source data and the structured claim-audit matrix are provided in the supplementary source-data archive. Analysis scripts, software version records, gene set definitions and resource snapshots are available from the corresponding author upon reasonable request.

## Figures and Tables

**Figure 1:**
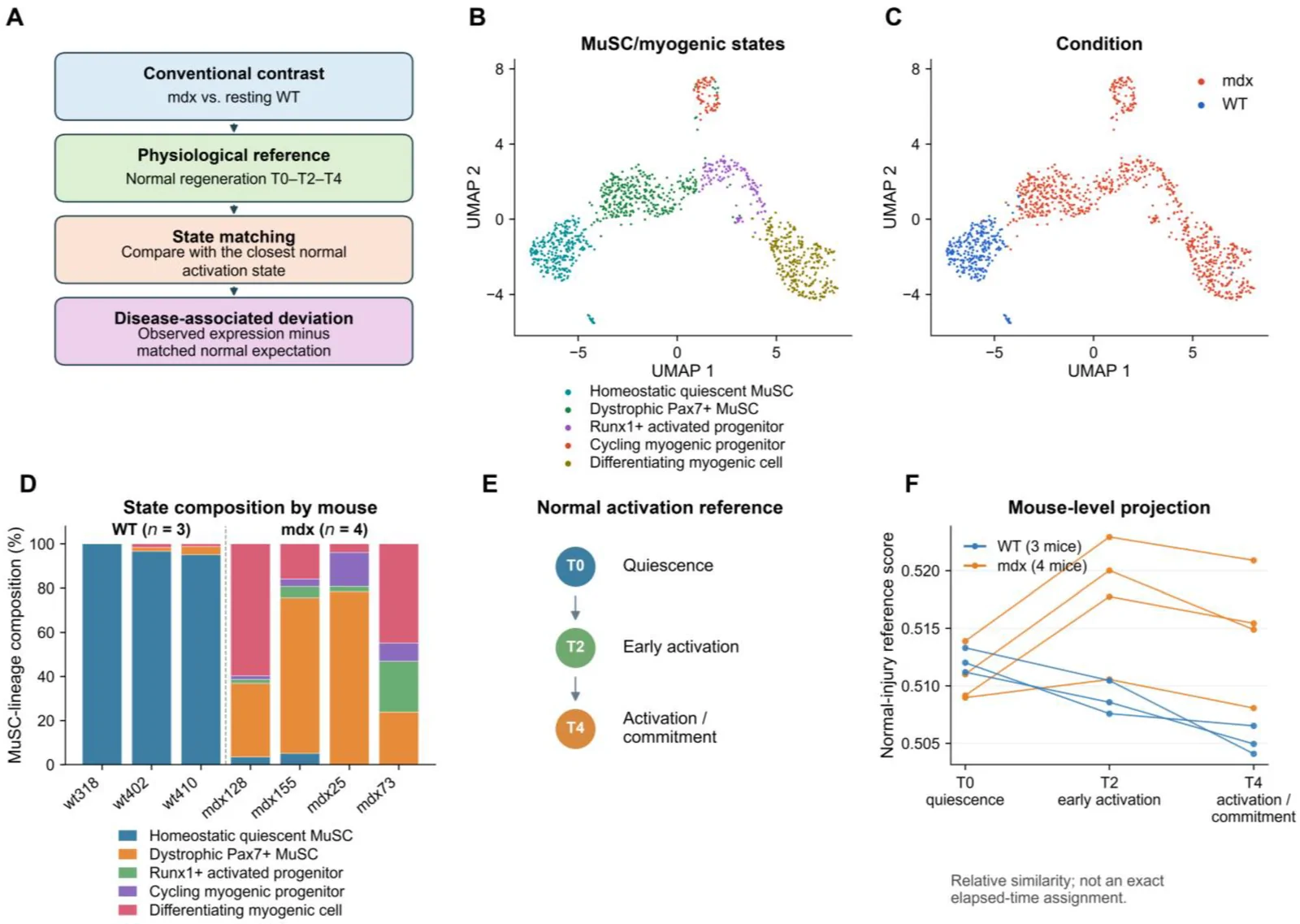
**Physiological reference mapping separates normal activation state displacement from disease-associated transcriptional deviation in dystrophic muscle stem cells.** (**A**) A conceptual workflow. A conventional comparison of mdx muscle stem cells (MuSCs) with homeostatic/quiescent wild-type (WT) MuSCs may combine transcriptional changes associated with normal activation and changes not explained by physiological regeneration. A normal regeneration reference was therefore used to match dystrophic cells to the most transcriptionally similar normal activation state. Disease-associated deviation was defined conceptually as the component of the observed dystrophic state remaining after accounting for the matched normal expectation. (**B**) Uniform manifold approximation and projection (UMAP) representation of the PRJNA771932 MuSC/myogenic compartment annotated into homeostatic quiescent MuSCs, dystrophic Pax7-positive MuSCs, Runx1-positive-activated progenitors, cycling myogenic progenitors and differentiating myogenic cells. Each point represents one nucleus; UMAP positions are descriptive and were not treated as independent biological replicates. (**C**) The same UMAP embedding colored by experimental condition, showing the distribution of WT and mdx nuclei across the MuSC/myogenic state space. (**D**) MuSC-lineage state composition for each biological replicate in PRJNA771932. Bars represent individual mice and show the percentage of MuSC-lineage nuclei assigned to each annotated state. The dataset contained three WT and four mdx mice. (**E**) Normal MuSC activation reference derived from GSE163856, represented by the T0 quiescent, T2 early-activation and T4 activation/commitment transcriptional signatures. These reference points define relative physiological states and should not be interpreted as exact elapsed-time assignments for dystrophic cells. (**F**) Projection of each PRJNA771932 biological replicate onto the normal T0–T2–T4 activation reference. Lines connect the three reference similarity scores calculated for each mouse; blue lines indicate WT mice and orange lines indicate mdx mice. WT samples showed the greatest relative similarity to the quiescent T0 reference, whereas several mdx samples showed greater relative similarity to the T2 or T4 activation references. These projections measure relative transcriptional similarity and do not assign an exact injury time or pseudotime to individual mice.

**Figure 2:**
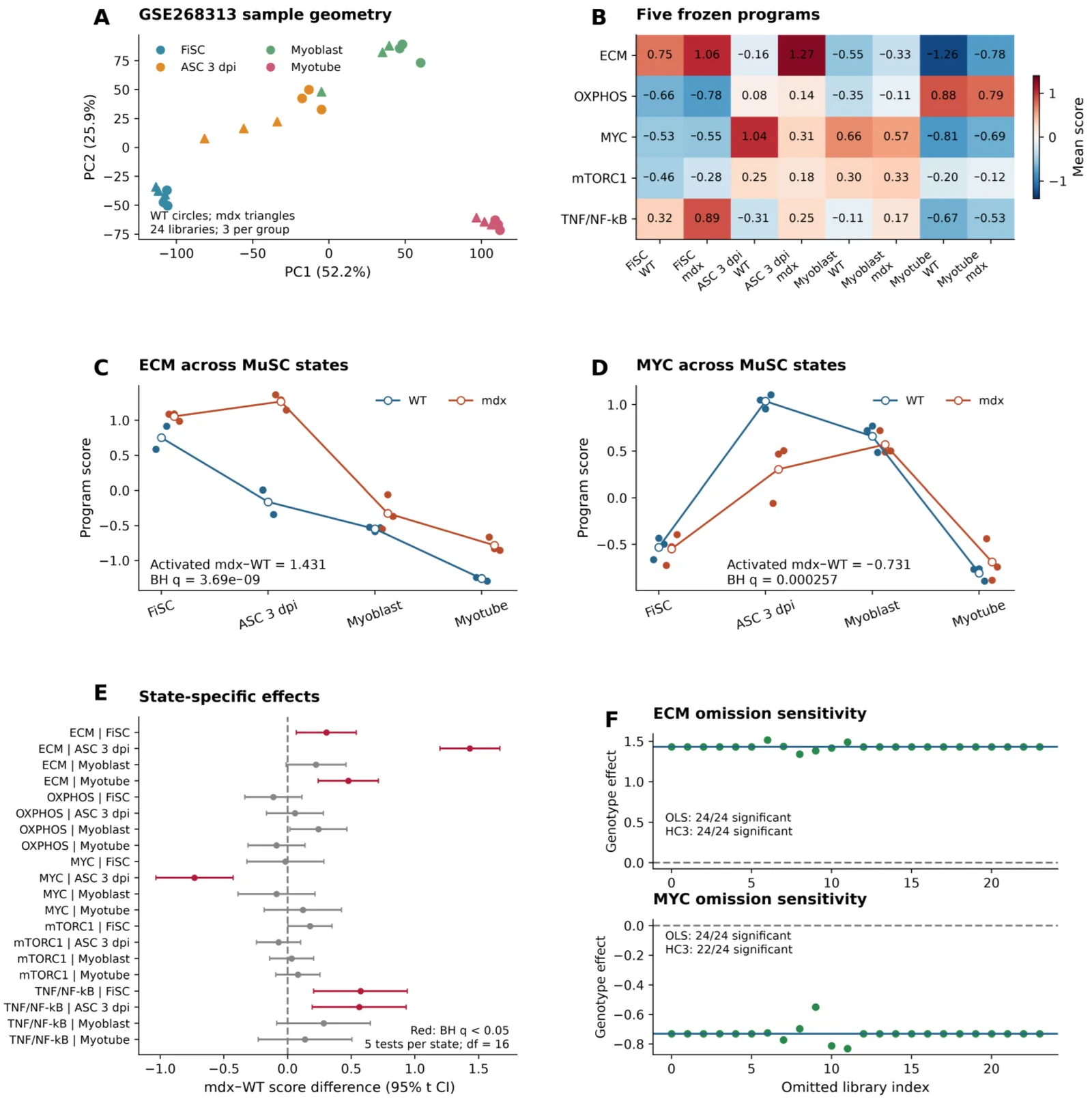
**Replicated genotype-by-state analysis distinguishes excessive, insufficient and state-dependent molecular responses across the dystrophic muscle stem cell lineage.** (**A**) Principal component analysis of normalized bulk RNA-sequencing profiles from GSE268313. The experiment comprised 24 libraries representing wild-type (WT) and mdx cells across four MuSC-lineage states, freshly isolated satellite cells (FiSC), activated satellite cells collected 3 days after injury (ASC 3 dpi), myoblasts and myotubes with three biological replicates per genotype–state group. Colors indicate cell state and symbols indicate genotype. PC1 and PC2 explain 52.2% and 25.9% of the total variance, respectively, demonstrating that lineage state is the principal source of transcriptomic variation. (**B**) Mean standardized scores for five prespecified molecular programs across the eight genotype–state groups. Columns are ordered along the MuSC-lineage progression, with WT and mdx shown separately within each state. Rows represent extracellular matrix (ECM), oxidative phosphorylation (OXPHOS), MYC targets, mTORC1 signaling and TNF/NF-κB signaling. Red indicates a higher program score and blue a lower score; values within cells are group means across three biological replicates. (**C**) ECM program trajectory across the four MuSC-lineage states. Filled points represent individual biological replicates and open circles connected by lines represent genotype-specific group means. Activated mdx MuSCs showed excessive ECM induction relative to state-matched WT cells (mdx−WT = +1.431, FDR = 3.69 × 10^−9^). (**D**) MYC program trajectory shown using the same representation as in panel C. MYC induction during activation was attenuated in mdx MuSCs, producing a negative activated-state residual relative to WT (mdx−WT = −0.731, BH q = 0.000257). Lines connect group means across states and do not imply that biological replicates were longitudinally paired. (**E**) State-specific mdx−WT residual estimates for all five programs. Points represent genotype-effect estimates from the prespecified genotype-by-state models, and horizontal bars indicate 95% t-based confidence intervals (16 residual degrees of freedom). Red denotes effects passing the BH threshold across five programs within each state of FDR < 0.05; gray denotes effects for which a statistically supported residual was not established. The vertical dashed line indicates no mdx−WT difference. (**F**) Leave-one-library-out sensitivity analysis for the activated-state ECM and MYC residuals. Each point represents the genotype-effect estimate obtained after omitting one of the 24 libraries. Horizontal blue lines indicate the corresponding full-data estimates, and dashed gray lines indicate no genotype difference. All leave-one-library-out analyses retained the original effect direction and FDR < 0.05, demonstrating that neither result was driven by a single library. All standard-model omission fits retained the effect direction and BH q < 0.05. HC3 significance persisted in 24/24 ECM and 22/24 MYC fits.

**Figure 3:**
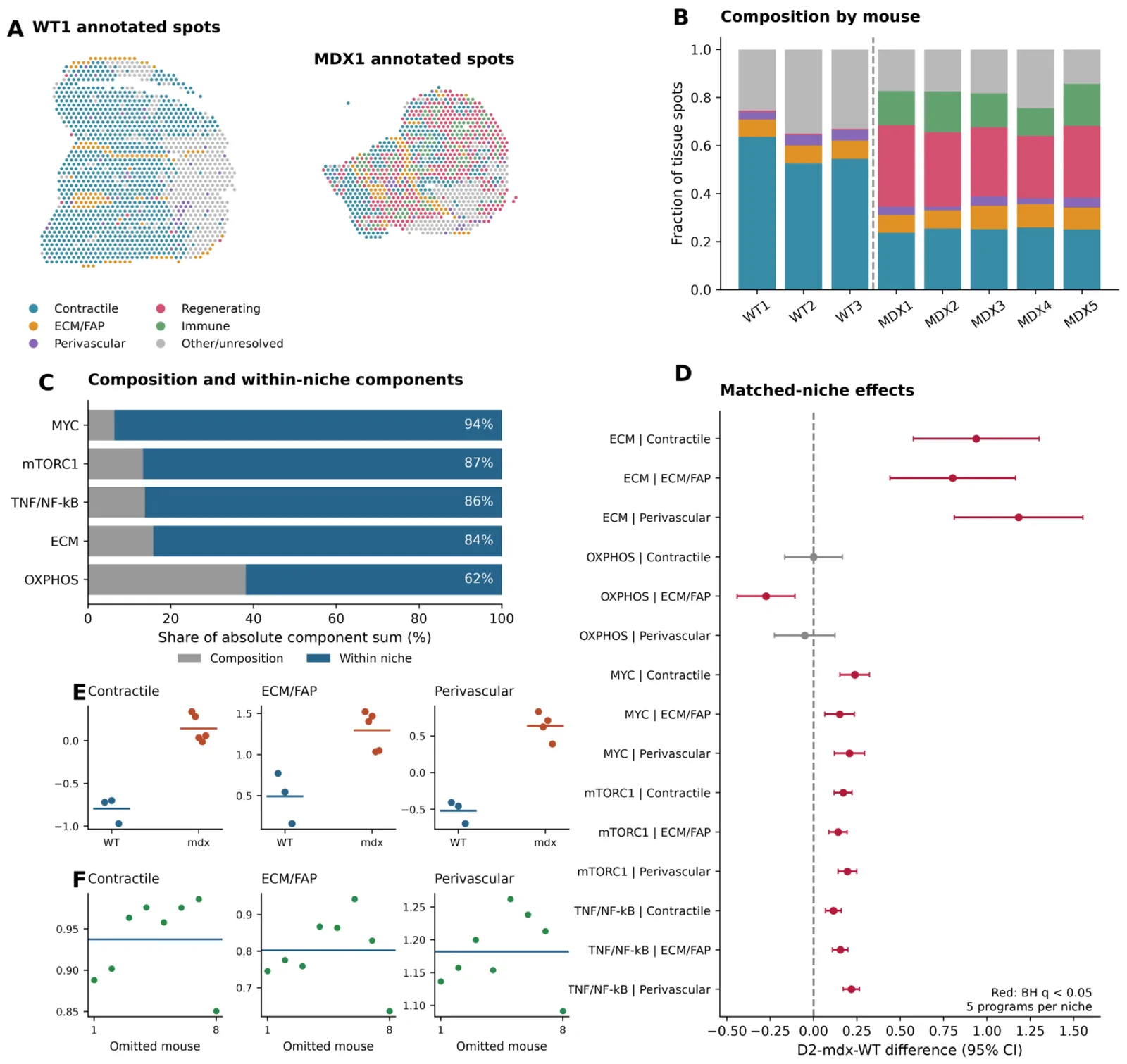
**Spatial niche matching separates altered tissue composition from disease-associated transcriptional remodeling within matched niches.** (**A**) Representative spatial spot-coordinate maps from GSE225766 showing wild-type DBA/2J and D2-mdx muscle annotated using the harmonized spatial niche classification. Maps were redrawn from source coordinates without the histology background. Colors indicate contractile-, ECM/FAP-, perivascular-, regenerating-, and immune-enriched spot niches; clusters not meeting the prespecified cross-replicate validation criteria are shown as other/unresolved. Regenerating- and immune-enriched niches were reproducibly detected in mdx tissue but lacked corresponding evaluable WT regions and were therefore retained for descriptive visualization but excluded from matched-niche genotype inference. (**B**) Spatial niche composition for each biological replicate. Bars show the fraction of tissue spots assigned to each niche in three WT and five D2-mdx mice; the dashed vertical line separates genotypes. Dystrophic tissue showed pronounced compositional remodeling, including expansion of regenerating- and immune-enriched regions and a reduction in the relative contractile compartment. (**C**) Decomposition of each observed tissue-level D2-mdx–WT program score contrast into contributions from differences in niche composition and transcriptional remodeling within niches shared between genotypes. Bars show the absolute contribution of each component relative to the total contribution. Within-niche remodeling accounted for 84% of the ECM contrast, 87% of mTORC1, 94% of MYC, 86% of TNF/NF-κB, and 62% of OXPHOS. These percentages partition the total contribution and are not estimates of variance explained. (**D**) A forest plot of D2-mdx–WT program score differences within the three cross-group-validated niches. Points represent estimated genotype differences and horizontal lines indicate 95% confidence intervals. Red points denote effects passing the within-niche false discovery rate threshold (FDR < 0.05); gray points indicate effects for which a corrected genotype difference was not established. The vertical dashed line indicates no genotype difference. Inference was performed using mouse × niche summaries as biological units rather than individual spatial spots. (**E**) Mouse-level ECM program scores within contractile-, ECM/FAP-, and perivascular-enriched niches. Each point represents one biological replicate after aggregation of eligible clusters within the corresponding mouse and niche; horizontal lines indicate genotype means. ECM was elevated in D2-mdx muscle within the contractile niche (D2-mdx–WT = 0.938, FDR = 0.00105), ECM/FAP niche (0.803, FDR = 0.00273), and perivascular niche (1.182, FDR = 0.000389). Contractile and ECM/FAP analyses included three WT and five D2-mdx mice; the perivascular analysis included three WT and four evaluable D2-mdx mice. Blue indicates WT and orange-red D2-mdx. (**F**) Leave-one-mouse-out sensitivity analysis of the ECM genotype effect within each matched niche. Each point represents the D2-mdx–WT estimate after omission of the indicated mouse; horizontal blue lines show the corresponding full-data estimates and dashed gray lines indicate no genotype difference. Forest intervals use the retained mixed-model degrees of freedom (df = 6); BH correction covers five programs per niche. Omission indices in F follow the accompanying source table.

**Figure 4:**
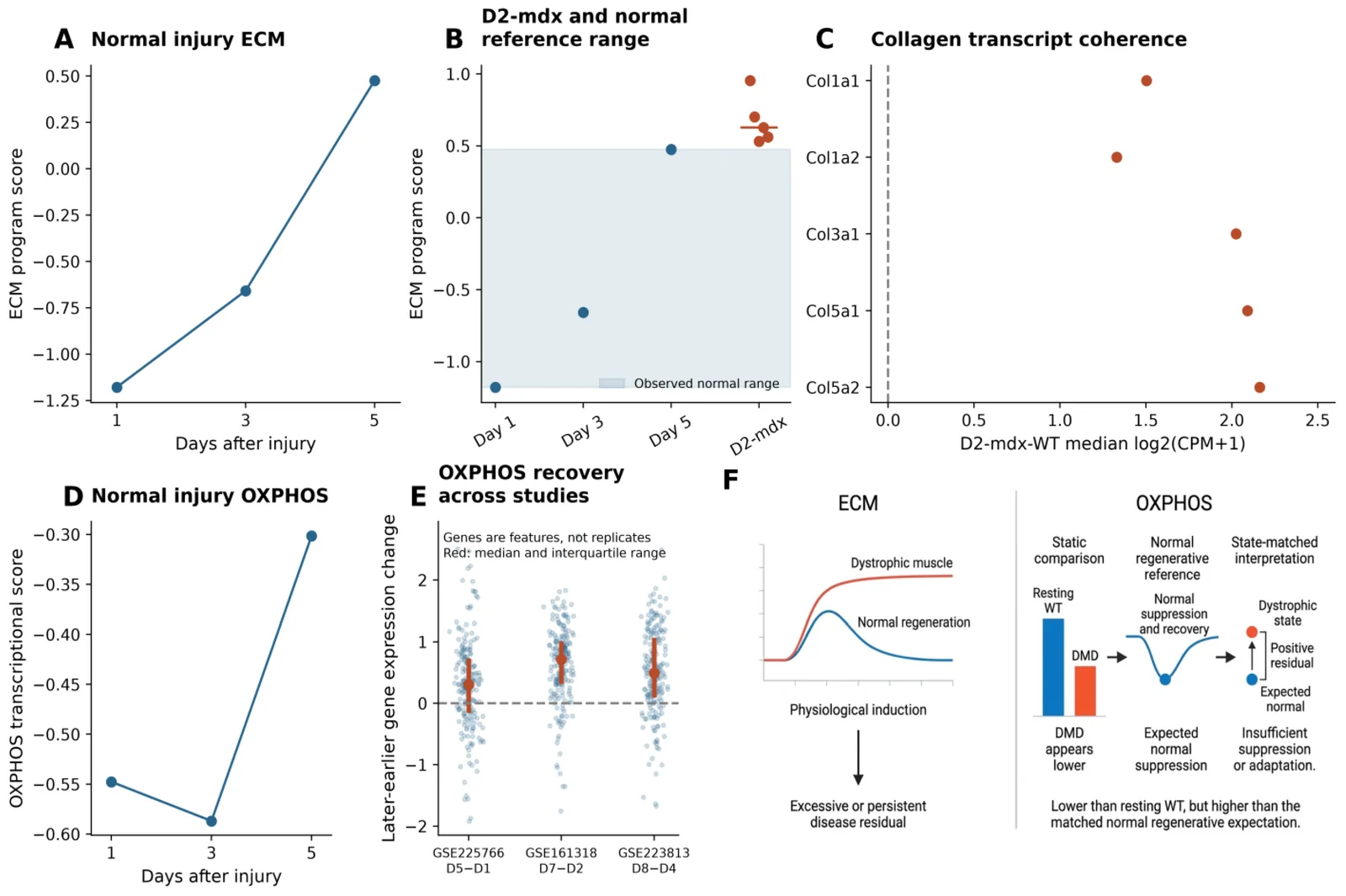
**Physiological injury references distinguish normal regenerative responses from disease-associated deviations.** (**A**) ECM program scores in the regeneration-enriched spatial niche of normal injured muscle at days 1, 3, and 5 after injury in GSE225766. ECM increased progressively across the sampled early regenerative interval. Because one section was available at each injury day, this trajectory is descriptive. (**B**) The position of five D2-mdx mice (mdx on the DBA/2J background) relative to the GSE225766 normal-injury ECM trajectory. The blue shaded band spans the minimum and maximum of the three sampled normal injury scores; it is neither a confidence interval nor a computed subtraction residual; individual D2-mdx mouse scores are shown in orange-red, with the horizontal line indicating the median. Dystrophic ECM scores exceeded the observed normal-injury range. (**C**) Three WT and five D2-mdx mice were compared for gene-level coherence of the frozen five-collagen ECM program in replicated whole-section GSE225766 samples. Points show the D2-mdx–WT difference in median log2(CPM + 1) for *Col1a1*, *Col1a2*, *Col3a1*, *Col5a1*, and *Col5a2*. All five D2-mdx mice were above the WT median for each collagen gene, with BH-adjusted FDR = 0.037. This whole-section analysis supports coordinated collagen induction but does not separate changes in tissue composition from within-niche regulation. (**D**) Normal-injury OXPHOS program trajectory in the regeneration-enriched niche of GSE225766. OXPHOS remained suppressed at days 1–3 and increased by day 5, consistent with early suppression followed by recovery. The trajectory is descriptive because one section was available per injury day. (**E**) Later-minus-earlier changes among measurable OXPHOS genes in three independent normal-injury spatial datasets: GSE225766, day 5 minus day 1; GSE161318, day 7 minus day 2; and GSE223813, day 8 minus day 4. Pale points represent individual genes; red points and bars indicate the median and interquartile range. The positive central tendency across datasets supports recovery of the OXPHOS program during regeneration. (**F**) A conceptual summary of physiological calibration. ECM induction occurs during normal regeneration, but excessive magnitude, persistence, or localization constitutes a disease-associated residual. The OXPHOS schematic illustrates how reduced expression relative to resting muscle may nevertheless represent insufficient suppression relative to normal regeneration. This interpretation is exploratory; the curves are illustrative rather than quantitative. The y axis in B reports program scores. D2-mdx is the disease cohort available in the spatial dataset. Tests in C are the saved mouse-level Wilcoxon tests with BH across five collagens; panels A, B, D and E are descriptive. OXPHOS refers only to transcription.

**Figure 5:**
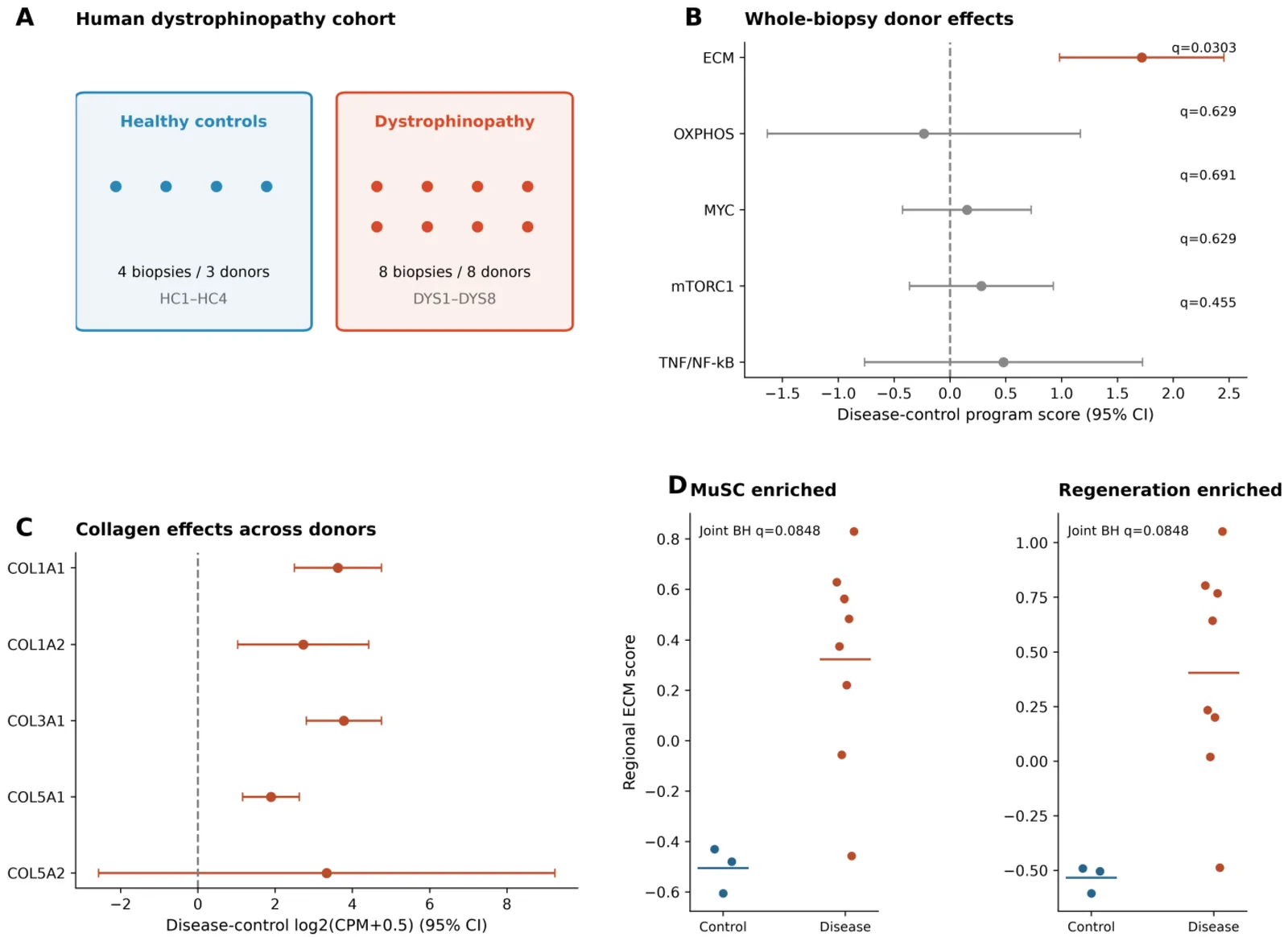
**Whole-biopsy and regional ECM transcription in human dystrophinopathy.** (**A**) An overview of the human spatial–transcriptomic cohort, comprising three control and eight dystrophinopathy donors. Two biopsies from one control donor were averaged so that each donor contributed one value. (**B**) Whole-biopsy differences in the five prespecified transcriptional programs between dystrophinopathy and control samples. Points indicate dystrophinopathy-minus-control program score differences. Bars show approximate Welch 95% confidence intervals. ECM was the only program significant after multiple-testing correction (effect = 1.718; q = 0.0303). Red indicates q < 0.05; the gray line indicates no difference. (**C**) Expression differences of five collagen genes contributing to the ECM program: COL1A1, COL1A2, COL3A1, COL5A1 and COL5A2. Points show mean log2(CPM + 0.5) differences, with approximate Welch 95% confidence intervals. All five genes were elevated in dystrophinopathy (q = 0.00758–0.0121). (**D**) ECM scores in MuSC-enriched and regeneration-enriched regions. Each point represents one donor; horizontal lines indicate group means. Scores were higher in dystrophinopathy, but neither regional comparison reached significance after correction (differences = 0.828 and 0.938, respectively; both q = 0.0848). *p* values were obtained by exact donor-label permutation tests, with Benjamini–Hochberg correction across five programs in B, five genes in C and ten region–program comparisons in D.

**Figure 6:**
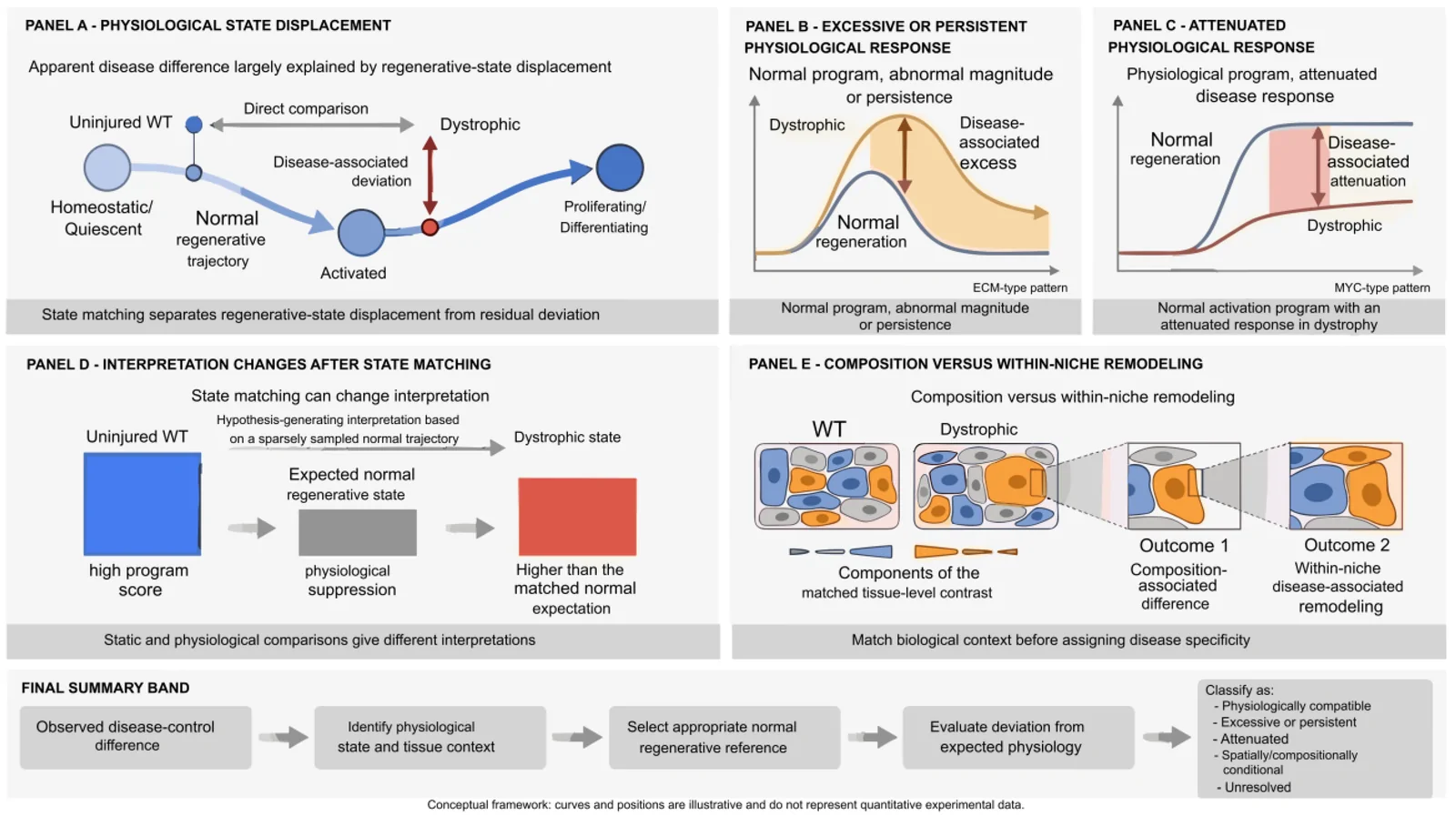
**Physiological reference mapping distinguishes regenerative state displacement from context-dependent disease-associated deviations.** (**A**) Conventional comparison of dystrophic muscle with uninjured wild-type (WT) muscle can conflate displacement along the normal regenerative trajectory with disease-associated dysregulation. Matching the dystrophic sample to the appropriate physiological state separates regenerative state displacement from the remaining disease-associated deviation. (**B**) An ECM-type response illustrates a physiological program that is normally transient but becomes excessive in magnitude or persistent in dystrophy. (**C**) A MYC-type response illustrates preservation of the expected activation-associated direction with an attenuated transcriptional response in dystrophy. (**D**) State matching can change the interpretation of a disease–control difference. Although a dystrophic OXPHOS program score may be lower than that of resting WT muscle, it may remain higher than the expected score for the matched regenerative state, suggesting insufficient suppression or altered metabolic adaptation. This OXPHOS interpretation is hypothesis-generating because the normal trajectory was sparsely sampled. (**E**) Tissue-level disease contrasts may reflect changes in niche composition, disease-associated remodeling within matched niches, or both. The summary workflow shows how observed disease–control differences are assigned a physiological state and tissue context, matched to an appropriate normal regenerative reference, evaluated relative to expected physiology, and classified as physiologically compatible, excessive or persistent, attenuated, spatially or compositionally conditional, or unresolved. Curves, positions and tissue diagrams are conceptual illustrations and do not represent quantitative experimental data.

**Table 1:** Transcriptomic datasets, biological replication, and analytical roles in the physiological reference-mapping framework.

Dataset	Modality	Biological Replication	Role
PRJNA771932 [16]	Mouse snRNA-seq	WT *n* = 3; untreated mdx *n* = 4; e23AON-treated mdx *n* = 4	MuSC state discovery; ECM cellular sources; exploratory communication and treatment analyses
GSE163856 [17]	Normal mouse MuSC bulk + Smart-seq2	Bulk: *n* = 3–4 per 0–120-min time; Smart-seq2: 2 units per T0/T2/T4	Acute normal activation reference
GSE268313 [9]	Sorted mouse MuSC-lineage bulk RNA-seq	3 libraries per genotype/state; 24 total; cross-state pairing undocumented	Primary replicated genotype-by-state model
GSE268314 [9]	Mouse single-cell reference	One library per genotype-condition	State mapping only; descriptive residual discovery
GSE225766 [18]	Mouse spatial transcriptomics	WT *n* = 3; D2-mdx *n* = 5; injury landmarks descriptive	Replicated genotype-by-niche model
GSE161318 [19]; GSE223813 [20]	Independent normal-injury spatial series	Study-specific time points	Independent direction of normal ECM/OXPHOS behavior
GSE288958 [21]	Human DMD snRNA-seq	11 donors; tissue-matched primary subset	Human MuSC stress test
Heezen et al. human spatial dataset [10]	Human control and dystrophinopathy Visium	4 control biopsies from 3 donors; 8 disease biopsies from 8 donors; donor-level analysis	Assessment of ECM transcription in human dystrophinopathy
GSE297388 [22]	Mouse background comparison	One library per genotype-background group	Descriptive genetic-background stress test
GSE180771 [23]	Mouse bulk RNA-seq	4-week and 18-month sedentary: 3/group each; exercise: 2/group	Independent AAV9-CRISPR treatment context; exercise descriptive

Table abbreviations: snRNA-seq, single-nucleus RNA sequencing; scRNA-seq, single-cell RNA sequencing; MuSC, muscle stem cell; WT, wild type; DMD, Duchenne muscular dystrophy.

## Data Availability

The original contributions presented in this study are included in the article/Supplementary Material. Further inquiries can be directed to the corresponding author.

## Supplementary Materials

The following supporting information can be downloaded at: https://www.mdpi.com/article/10.3390/cells15191743/s1, File S1: Supplementary Methods S1–S21; Figure S1: Structured claim-audit workflow for physiological reinterpretation of published disease findings; Figure S2: Mouse lineage contributions to captured collagen transcripts; Figure S3: Exploratory ECM-lesion-composition association; Figure S4: Fixed regeneration control program; Figure S5: FOXO transcript expression; Figure S6: Inferred FOXO target-program scores; Figure S7: Exploratory immune satellite interaction scores; Figure S8: Exploratory exon skipping program comparisons; Figure S9: Transcriptional programs after AAV9 CRISPR correction; Figure S10: Structured claim-level audit of published dystrophic-muscle findings using physiological reference matching; Table S1: Structured claim-level audit of 79 claims from seven dystrophic-muscle publications.
