# Supplementary material for: Physiological Reference Mapping Reveals Context-Dependent Dysregulation of Regenerative Programs in Duchenne Muscular Dystrophy
Source: Cells. 2026 Sep 24;15(19):1743. doi: 10.3390/cells15191743 (PMC13664023; doi:10.3390/cells15191743)
Supplement: Supplementary file 1 [file cells-15-01743-s001.zip › cells-4555345-supplementary figures.pdf]

Physiological Reference Mapping Reveals Context-Dependent Dysregulation of Regenerative Programs in Duchenne Muscular Dystrophy.

Supplementary Data and Methods

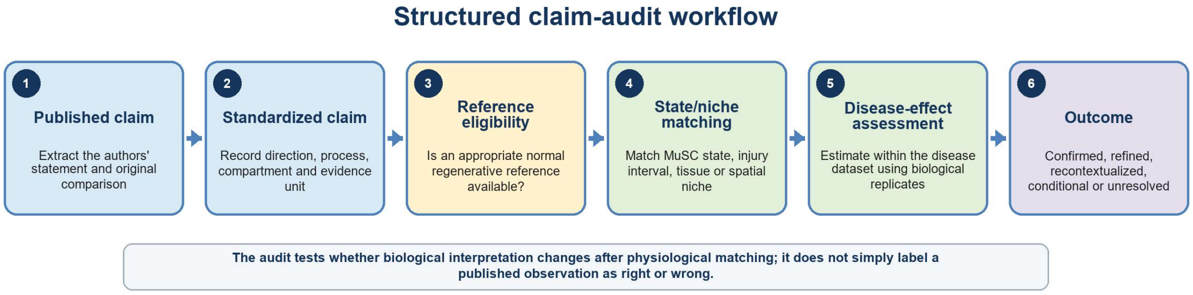

Supplementary Figure S1. Structured claim-audit workflow for physiological reinterpretation of published disease findings. Published claims were first extracted together with the comparison reported by the original authors. Each claim was then standardized by recording its direction, molecular process, biological compartment and evidence unit. Reference eligibility was assessed by determining whether an appropriate normal regenerative reference was available. Eligible claims were matched by MuSC state, injury interval, tissue or spatial niche, after which the disease effect was evaluated within the corresponding dataset using biological replicates. Each claim was assigned a prespecified outcome-confirmed, refined, physiologically recontextualized, spatially or compositionally conditional, or unresolved. This audit tests whether physiological matching changes the biological interpretation of a published observation; it does not classify the original observation as simply correct or incorrect.

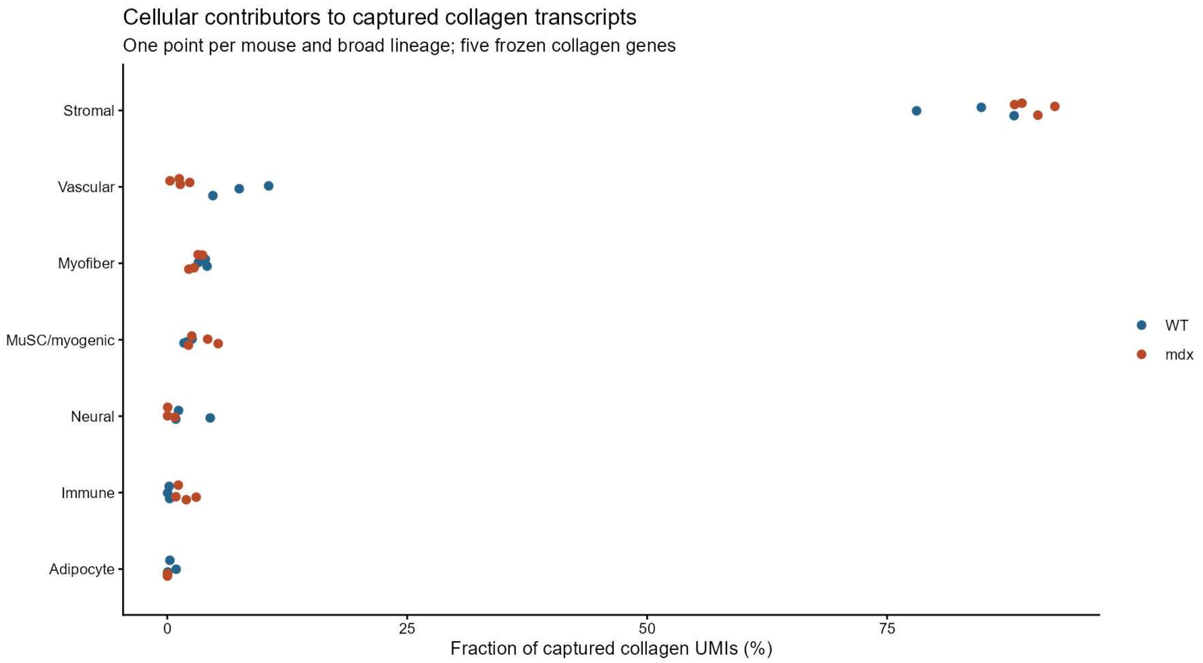

Supplementary Figure S2. Mouse lineage contributions to captured collagen transcripts. Each point is one mouse/lineage; percentages are contributions to captured counts for the five collagen genes, not tissue collagen burden. Three WT and four mdx mice. Source: ECM_captured_fraction_by_broad_lineage.csv. Cell-type tests and marker sensitivities are separate tables.

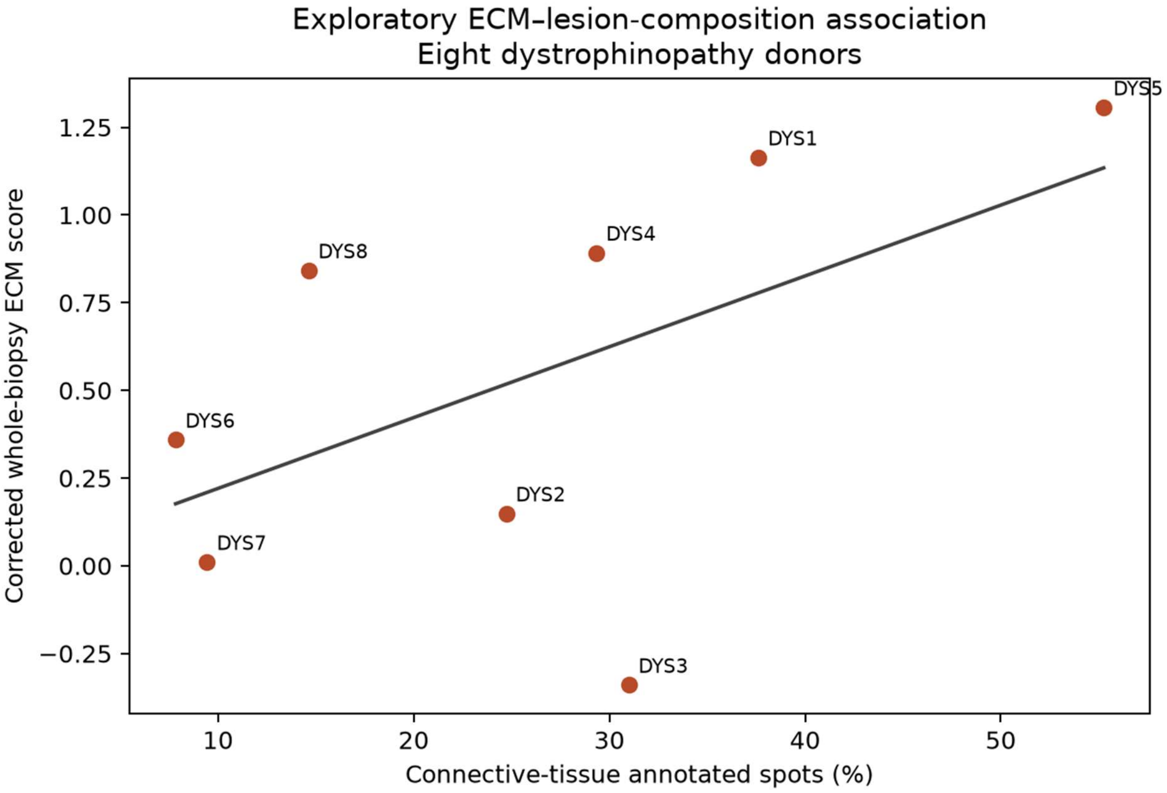

Supplementary Figure S3. Exploratory ECM-lesion-composition association. Each point is one dystrophinopathy donor; line is the fitted OLS trend. Eight donors; slope per 10 percentage points=0.202 (95% CI −0.110 to 0.513), Spearman rho=0.524, exact P=0.197. Annotation is not independent fibrosis measurement. Sources: ECM_connective_annotation_sample_data.csv, ECM_connective_annotation_association.csv.

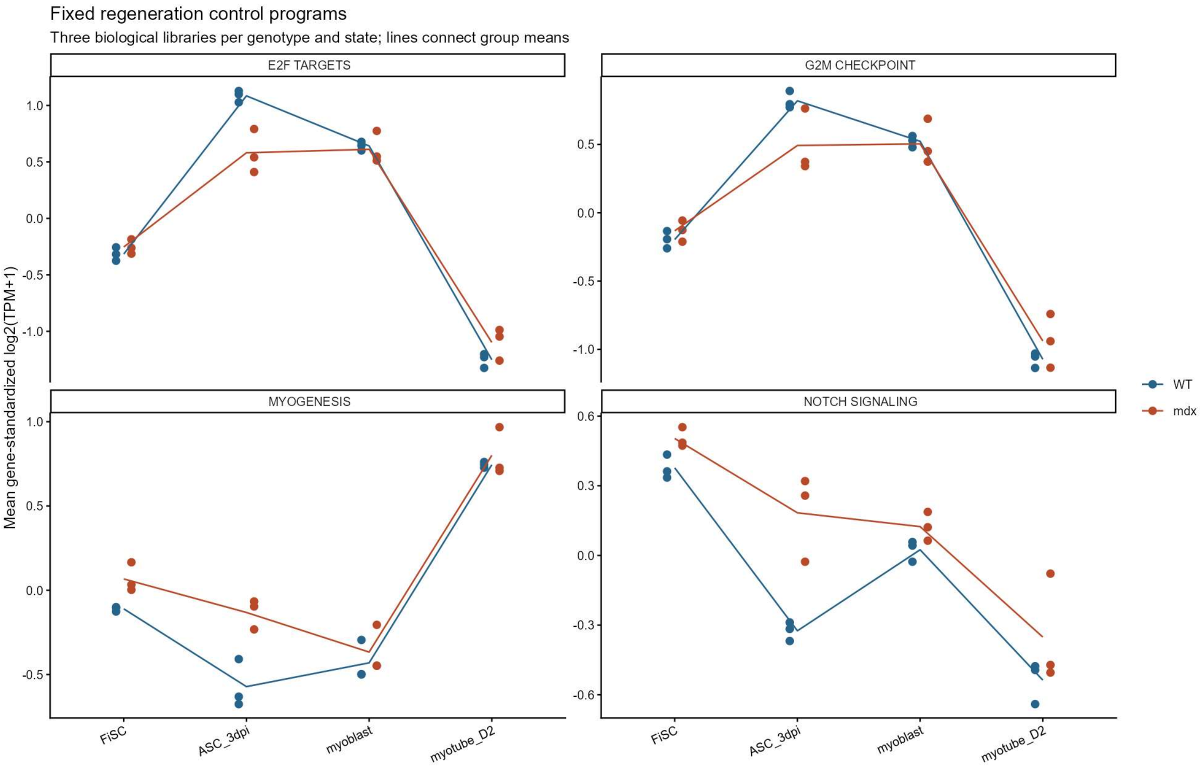

Supplementary Figure S4. Fixed regeneration control program. Points represent individual libraries (three per genotype/state); lines connect group means and do not indicate longitudinal pairing. Panels have separate y scales. Source: Regeneration_control_sample_scores.csv; full score and CAMERA contrast tables report effect sizes and BH families.

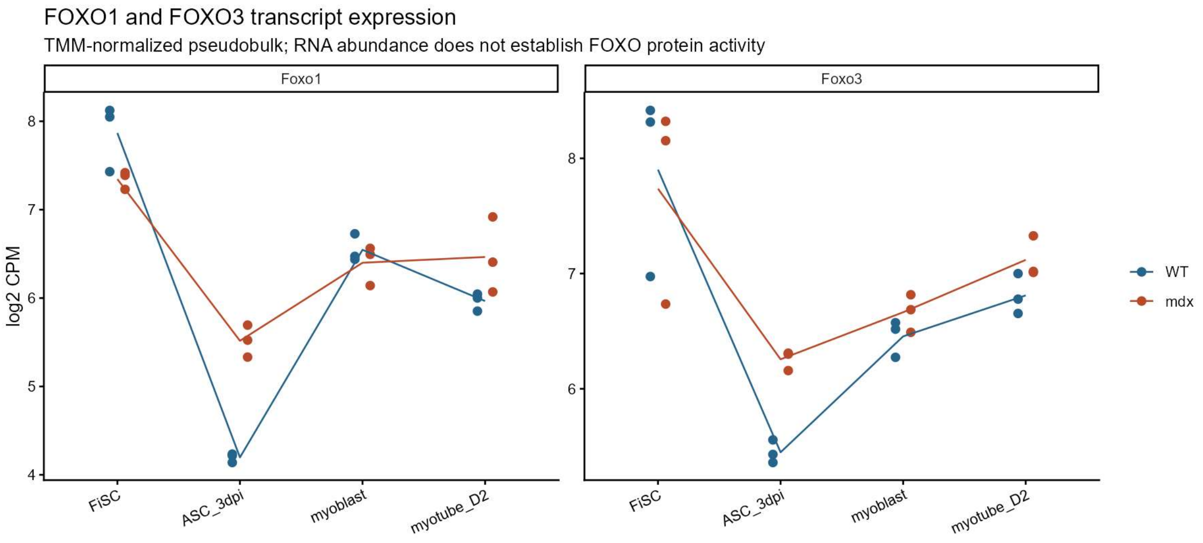

Supplementary Figure S5. FOXO transcript expression. Points are individual libraries; lines connect group means. TMM-normalized log2CPM expression is descriptive; inference uses the edgeR quasi-likelihood contrast table with BH across eight gene/state tests. Source: FOXO_transcript_sample_expression.csv and FOXO_transcript_edgeR_tests.csv.

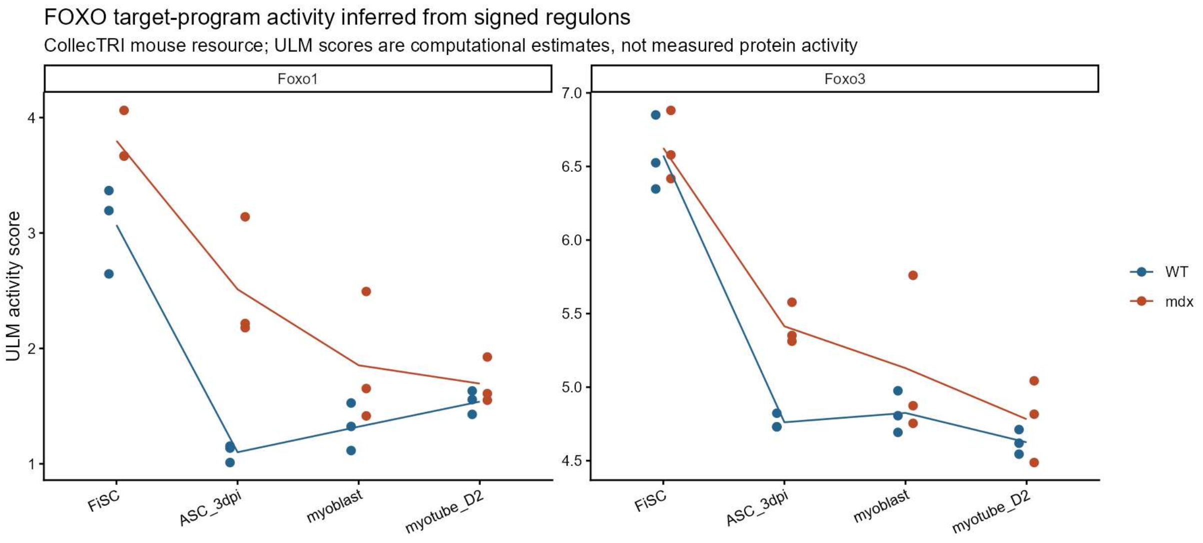

Supplementary Figure S6. Inferred FOXO target-program scores. Individual-library ULM scores from all signed measured targets; lines connect group means. Three libraries per genotype/state. Computational scores are not measured protein activity. Full state contrasts and unique-target sensitivity appear in FOXO_inferred_activity_state_tests.csv; plotted data in FOXO_inferred_activity_by_library.csv.

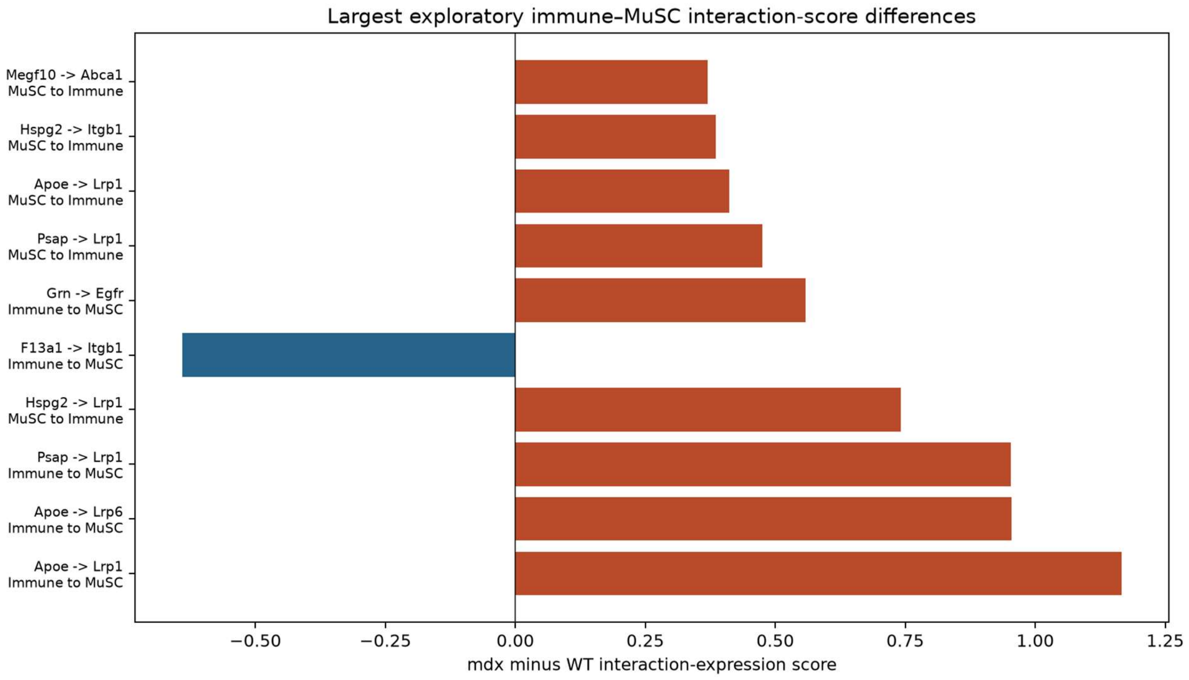

Supplementary Figure S7. Exploratory immune satellite interaction scores. Ten largest absolute interaction-expression differences are shown descriptively from 38 eligible directional tests. Bars are mdx−WT differences between mouse means, with three WT/four mdx mice. None survives BH correction (minimum q=0.136); bar ranking is not significance ranking. Sources: LIANA_mouse_level_disease_tests.csv, all per-mouse scores and sensitivity/coverage tables

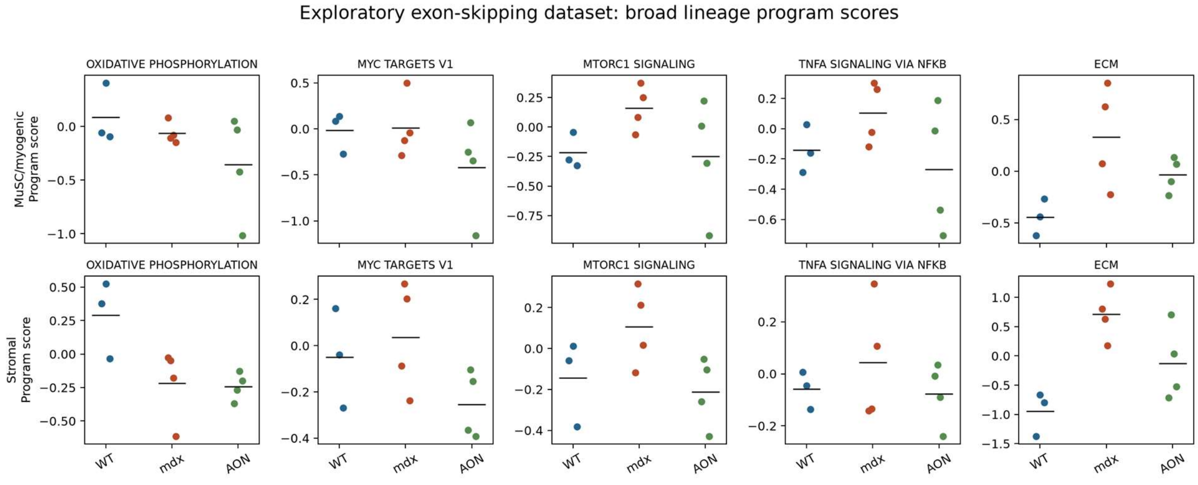

Supplementary Figure S8. Exploratory exon skipping program comparisons. Each point is one mouse; short horizontal lines indicate group means. Three WT, four mdx and four e23AON-treated mice. Five fixed programs in two validated broad compartments; axes use reference-standardized scores and separate panel scales. None of ten treated-mdx comparisons survives BH correction. AON denotes antisense oligonucleotide treatment. Sources: Supplementary_Figure_S8/Treatment_program_scores.csv, Treatment_program_contrasts.csv and annotation/QC tables.

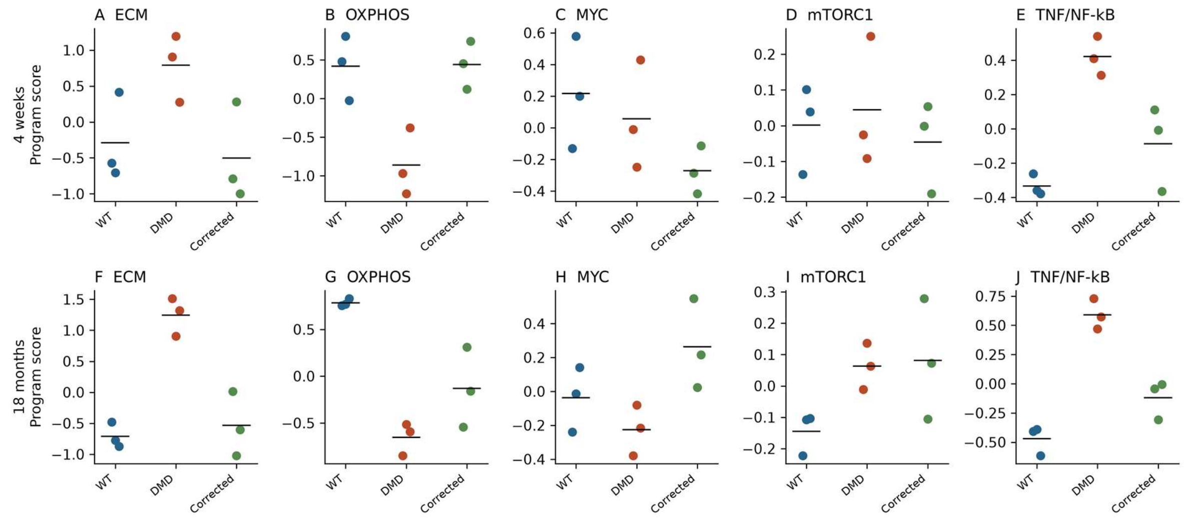

Supplementary Figure S9. Transcriptional programs after AAV9 CRISPR correction. Panels A–E show four-week sedentary mice; F–J show 18-month sedentary mice. Each point is one mouse (three/group); horizontal lines indicate group means. WT denotes wild type, DMD untreated delEx44, and Corrected AAV9-CRISPR/Cas9-treated delEx44. Columns show the five original programs. Scores are mean standardized gene expression within each cohort; axis scales vary by panel. Corrected−DMD score-model BH q values are 0.0624, 0.0208, 0.281, 0.530 and 0.0208 for A–E, and 0.00533, 0.0779, 0.0559, 0.860 and 0.00533 for F–J. Correction covers ten program/cohort tests per contrast. The four score-model discoveries have concordant CAMERA support. Exact two-sided tests have minimum attainable P=0.10 with three mice/group and yield no significant results. These are whole-muscle transcriptional measurements. Sources: GSE180771 scores.csv, contrasts.csv, camera.csv and coverage.csv; methods in S21.

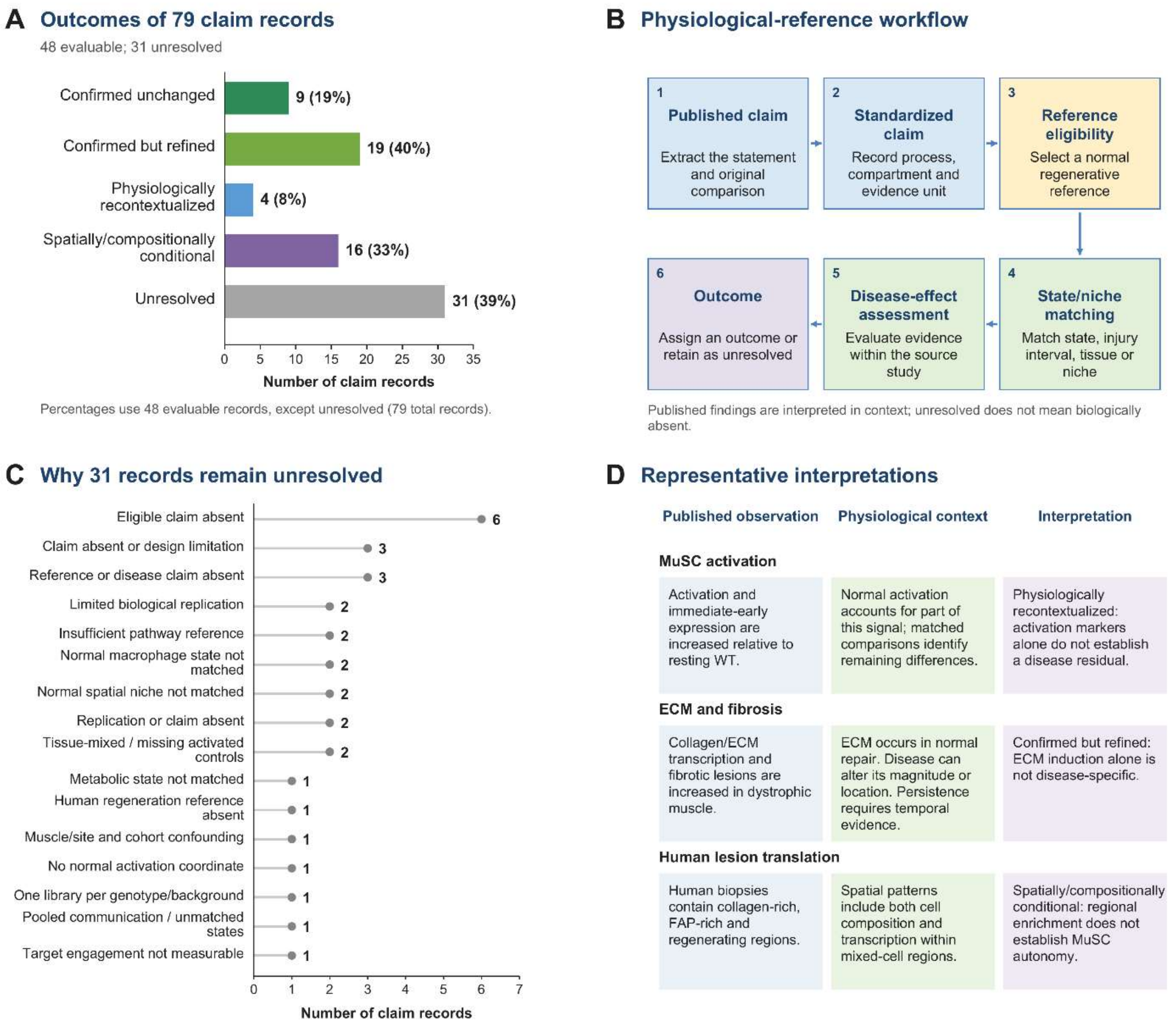

Supplementary Figure S10. Structured claim-level audit of published dystrophic-muscle findings using physiological reference matching. The audit contains 79 claim records from seven dystrophic-muscle publications. (A) Distribution of outcomes. Of 48 evaluable records, 19 (40%) were confirmed but refined, 16 (33%) were spatially or compositionally conditional, nine (19%) were confirmed unchanged, and four (8%) were physiologically recontextualized. The remaining 31 records were unresolved (39% of all 79 records). (B) Workflow for extracting claims, identifying suitable physiological references, matching state or niche, assessing source-study evidence and assigning outcomes. Published observations were interpreted in context rather than judged simply correct or incorrect. (C) Recorded reasons for unresolved outcomes, including missing claims or references, inadequate replication, unmatched states or niches, cohort confounding and measurement limitations. Unresolved outcomes do not indicate biological absence. (D) Representative interpretations concerning MuSC activation, ECM and fibrosis, and human lesion organization. ECM induction alone is not disease-specific; regional enrichment does not establish MuSC-autonomous production. Full source citations, eligibility decisions and justifications are provided in Supplementary Table S1.
