# Supplementary material for: Physiological Reference Mapping Reveals Context-Dependent Dysregulation of Regenerative Programs in Duchenne Muscular Dystrophy
Source: Cells. 2026 Sep 24;15(19):1743. doi: 10.3390/cells15191743 (PMC13664023; doi:10.3390/cells15191743)
Supplement: Supplementary file 1 [file cells-15-01743-s001.zip › File S1-cells-4555345-Supplementary Method S1-S21 .pdf]

Supplementary Methods

Literature citations use the reference numbering in the main manuscript.

These methods expand the concise workflow described in the main manuscript. Sections S1-S3 define the analysis architecture, computational environment, and inferential rules; Sections S4-S10 describe dataset-specific processing, physiological matching, spatial analysis, and human validation; Sections S11-S13 define structured claim auditing, multiplicity control, reporting requirements, and interpretation boundaries; Section S14 summarizes dataset roles and biological replication. Sections S15–S20 describe the additional ECM source, lesion-composition, regeneration-control, FOXO, communication and treatment analyses. Figures S2–S8 and their machine-readable tables report the added analyses, including negative results. Section S21 and Figure S9 describe the independent AAV9-CRISPR/Cas9 treatment analysis.

S1. Objective and analysis architecture

The study tested whether transcriptomic differences in dystrophic muscle and dystrophic muscle stem cells (MuSCs) remained abnormal after matching the relevant normal regenerative state and spatial niche. Analyses were separated into discovery, mapping, inferential eligibility, frozen validation, and sensitivity stages. A dataset could contribute to state mapping without being eligible for genotype inference. The regeneration-reference framework was defined as $Y_{\mathrm{DMD}}(\mathrm{c}) = \mu_{\mathrm{normal}}(\mathrm{c}) + D_{\mathrm{DMD}}(\mathrm{c})$, where $Y_{\mathrm{DMD}}(\mathrm{c})$ is the observed dystrophic program in context c, $\mu_{\mathrm{normal}}(\mathrm{c})$ is the expected normal program at the matched context, and $D_{\mathrm{DMD}}(\mathrm{c})$ is the context-dependent DMD-associated deviation. Context included MuSC state, regenerative interval, spatial niche, tissue, genetic background, species, and experimental platform. Cross-study raw scores were never pooled or directly subtracted. Replicated disease datasets estimated residuals; independent normal datasets calibrated the expected state, direction, or range.

Five safeguards were applied: biological animals, donors, or independent libraries were statistical units; cells, nuclei, spots, and clusters were not treated as independent replicates; state/niche mappings were frozen independently of pathway outcomes where possible; definitions and decision thresholds were frozen before confirmatory tests; and analyses were permitted to return unresolved when replication, correspondence, tissue matching, or resolution was inadequate.

S2. Project organization and software

Public raw or author-processed files were stored under data; intermediate matrices and serialized objects under processed or dataset-specific directories; R programs under scripts; machine-readable outputs under results/tables; figures under results/figures; and frozen plans, eligibility decisions, and narrative checkpoints under docs.

Exact software and package versions are reported in the main Methods under Software and reproducibility. Complete session information and supporting reproducibility records are available from the corresponding author upon reasonable request.

S3. Statistical units and eligibility

Inferential eligibility required identifiable biological units and compatible states or niches. Primary mouse comparisons required at least three biological units per group; one-library or one-section references were descriptive. Human spatial tests used three control and eight disease donors after averaging HC1/HC3 within their shared donor, not unrestricted biopsy-label permutations. Cross-state pairing of bulk libraries was undocumented; the independent-library assumption is therefore a limitation. Small cohorts and sensitivity analyses do not establish population representativeness.

The mouse, human donor, or independent bulk library was the analysis unit, according to the design of the source dataset. Donor identity was retained when multiple biopsies originated from the same individual. Individual cells, nuclei, spots, pseudotime bins, and computational clusters were never counted as independent biological replicates. Cell-level generalized additive models were explicitly labeled descriptive.

S4. PRJNA771932 replicated mouse single-nucleus workflow

The processed Cell Ranger archive was obtained from Zenodo rather than reconstructing more than 100 GB of raw SRA data. The downloaded archive was 1,479,948,358 bytes with MD5 4928647F8D04416874FCD720CCA76723. WT (n=3) and untreated mdx (n=4) animals were retained; the four exon-skipping-treated mdx samples were subsequently analyzed in the exploratory extension described in S20.

Matrices were imported independently by mouse. Nuclei required at least 300 detected genes, mitochondrial RNA no greater than 20%, and log10 genes per UMI at least 0.80. No universal upper feature/count threshold was imposed. scDblFinder was run within each mouse so that biological differences between animals were not interpreted as doublets.

Filtered objects were merged without batch integration. Counts were log-normalized, 3,000 highly variable genes selected, mitochondrial percentage regressed during scaling, and 50 PCs computed. The first 30 PCs were used for neighbors and UMAP. Clustering was examined at resolutions 0.2, 0.4, 0.6, and 0.8; resolution 0.4 produced 18 broad clusters and was used for canonical-marker annotation.

The satellite-cell and activated-myogenic clusters were selected, yielding 1,089 MuSC/myogenic nuclei. The subset was reprocessed with 2,000 variable genes, 30 computed PCs, the first 20 PCs, and resolution 0.4.

Five states were annotated from marker programs: WT homeostatic/quiescent, dystrophic Pax7-positive, Runx1-positive activated, cycling, and differentiating. Annotation preceded frozen pathway interpretation.

Raw counts were summed by mouse for whole-lineage pseudobulk and by mouse-state for state-resolved analyses. The edgeR model used condition as the explanatory variable; filterByExpr, TMM normalization, dispersion estimation, and quasi-likelihood F tests were applied. Positive log2 fold-change denotes higher mdx expression. Mouse-state summaries required at least 10 nuclei and were descriptive when a state lacked representation in both genotypes.

An mdx-only constrained trajectory used 804 nuclei ordered from dystrophic Pax7-positive through activated, cycling, and differentiating states. State centroids were calculated in the first 15 PCs and connected in that specified order. Each nucleus was projected to the nearest path point and assigned pseudotime from 0 to 1. Nucleus-level GAMs were descriptive. Regulator activity was summarized in mouse-by-pseudotime-bin pseudobulks requiring at least eight nuclei.

S5. Normal acute MuSC reference: GSE163856

The replicated bulk reference profiled sorted Pax7-nGFP MuSCs at 0, 15, 30, 60, 90, and 120 minutes. Biological sample counts were 4, 4, 3, 4, 3, and 4. Normal-only time effects supplied trajectory feature selection, pathway direction, and leave-one-sample-out calibration; disease data were not used to select normal temporal genes.

The fixed Smart-seq2 experiment contained 280 Pax7-positive cells at T0, T2, and T4 hours, with two mouse or pooled-mouse units per time. Counts were pseudobulked by biological identifier. One-versus-rest edgeR contrasts generated time signatures; the 50 strongest positive genes per time were retained. PRJNA771932

cells were mapped using within-cell percentile ranks, reducing dependence on Smart-seq2 versus 10x scale. Closest-time assignments were relative similarities rather than elapsed-time labels.

S6. GSE268313 replicated genotype-by-state analysis

The dataset comprised 24 Salmon-quantified bulk RNA-seq libraries representing four MuSC-lineage states: freshly isolated satellite cells (FiSCs), MuSCs activated at 3 days post-injury (ASC_3dpi), cultured myoblasts, and day-2 myotubes. Each state included three independent biological replicates for both WT and mdx mice. Transcript-level estimated counts and transcripts per million (TPM) were aggregated using stable Ensembl gene identifiers, producing an unfiltered matrix of 53,392 genes.

Separate gene-filtering procedures were used for descriptive quality assessment and differential-expression modeling. For quality assessment, CPM values were calculated from the unrounded Salmon estimated counts. A total of 16,895 genes with CPM ≥1 in at least three libraries were retained for the filtered log2(CPM + 0.5) expression matrix and sample-correlation analysis. PCA was performed using the 5,000 most variable genes within this quality-control set. PC1 and PC2 explained 52.22% and 25.85% of the variance, respectively. Mean within-group correlations ranged from 0.9602 to 0.9930, and 21 of 24 libraries had a nearest neighbor from the same genotype–state group. No library was excluded.

Differential-expression modeling used a separate, group-aware filtering procedure. Salmon estimated counts were rounded to integers, while the original unrounded matrices were retained for descriptive analyses. The rounded counts were entered into an edgeR DGEList, and filterByExpr was applied using the eight genotype-by-state groups with min.count = 10. This procedure retained 22,319 genes for differential-expression analysis. The difference between the 16,895-gene quality-control set and the 22,319-gene modeling set reflects their distinct filtering criteria and analytical purposes.

TMM normalization, robust dispersion estimation, and quasi-likelihood F tests were performed using a single genotype-by-state design, with WT and FiSC as the reference levels. Fourteen prespecified contrasts evaluated WT state transitions, mdx-versus-WT differences within each state, and genotype-by-transition interactions. An omnibus three-degree-of-freedom test assessed whether the expression trajectory differed by genotype across any state transition. Pairing across states was not imposed because the available metadata did not establish that samples represented repeated measurements from the same animals.

Fifty mouse-mapped MSigDB Hallmark gene sets were obtained using msigdbr and evaluated with limma CAMERA, which accounts for inter-gene correlation. WT, mdx, and genotype-by-transition effects were assessed across the FiSC-to-ASC_3dpi, ASC_3dpi-to-myoblast, and myoblast-to-myotube transitions. Program-level outcomes were classified as preserved, attenuated but retained, excessive or persistent, reversed, failed or lost, disease-specific or redirected, or without a clear deviation.

S7. Frozen programs and matched MuSC model

Five programs were frozen before formal fitting: Col1a1/Col1a2/Col3a1/Col5a1/Col5a2 collagen-ECM; HALLMARK_OXIDATIVE_PHOSPHORYLATION; HALLMARK_MYC_TARGETS_V1; HALLMARK_MTORC1_SIGNALING; and HALLMARK_TNFA_SIGNALING_VIA_NFKB.

The primary MuSC model was pathway score ~ genotype * state. State-specific mdx-minus-WT contrasts and the omnibus interaction were multiple-testing corrected. Robustness comprised 24 leave-one-library-out refits and HC3 heteroscedasticity-consistent errors. Directionally stable but HC3-sensitive early TNF/NF-kB estimates were downgraded to provisional.

Scores averaged gene-standardized log2(TPM+1) values, with duplicate gene symbols collapsed by mean log expression. State-specific genotype tests use t intervals with 16 residual degrees of freedom. BH

correction is applied explicitly across five programs within each state, including each omission fit. The activated MYC BH value is 0.000257. Standard omission fits retain ECM/MYC significance in 24/24 runs; HC3 does so in 24/24 and 22/24, respectively.

Every program was decomposed to measured member genes. Coherence required distributed directional support, replicate stability, and absence of domination by one gene. Collagen conclusions required concordance across all five frozen collagens. OXPHOS, MYC, and mTORC1 received gene-level state/timing audits.

S8. GSE225766 spatial disease-residual workflow

Spot matrices were imported per section and filtered using predefined library-size, detected-feature, mitochondrial-fraction and in-tissue criteria recorded in the dataset-specific scripts. Normalization and unsupervised dimension reduction did not use genotype-specific pathway outcomes. Clusters were annotated using contractile, fibro-adipogenic/ECM, vascular/perivascular, immune and regenerative marker programs.

Composition and within-niche components were calculated using a symmetric two-factor decomposition of mouse-balanced group centroids across niches shared between genotypes. For each niche *k*, the composition component was $(p_{D,k} - p_{W,k})(\mu_{D,k} + \mu_{W,k})/2$, and the within-niche component was $(\mu_{D,k} - \mu_{W,k})(p_{D,k} + p_{W,k})/2$. Here, p denotes the niche fraction, μ denotes the niche program mean, and D and W denote D2-mdx and WT, respectively. Each component was summed across niches. The within-niche percentage was calculated as $100 \times |\text{within}|/(|\text{within}| + |\text{composition}|)$, expressing its contribution relative to the combined magnitudes of the two components. These percentages do not represent variance explained or causal mediation.

Cross-dataset niche correspondence used marker programs rather than label identity. Contractile, ECM/FAP and perivascular mappings were accepted only when marker direction and projection sensitivity were coherent. Niche definitions were fixed before testing the five pathways.

Cluster medians were reduced to one value per mouse, validated niche and pathway using spot-number-weighted means; unweighted means were used for sensitivity analyses. The final model input contained 115 unique mouse–niche–pathway observations from three WT and five D2-mdx mice. These observations represented repeated measurements within mice. The primary model included genotype, validated niche and their interaction, with a random intercept for mouse. Contractile and ECM/FAP comparisons included three WT and five D2-mdx mice; perivascular comparisons included three WT and four D2-mdx mice. Mixed-model contrasts used six denominator degrees of freedom, with Benjamini–Hochberg correction across five programs within each niche.

Models were refitted after leaving out each mouse. Robustness was assessed by examining whether effect direction and FDR < 0.05 were retained across the leave-one-mouse-out fits and under weighted and unweighted aggregation.

S9. Normal spatial calibration

GSE225766 normal injury Day 1, Day 3, and Day 5 sections were directly comparable internally but included one section per time point; above/below-range classifications were descriptive. GSE161318 notexin Day 2/5/7 and GSE223813 cardiotoxin Day 4/8 spatial plus Day 1/2/4 single-cell data were analyzed independently for normal temporal direction. Raw scores were never pooled across studies.

Prespecified questions were whether ECM showed transient induction and later resolution; whether OXPHOS showed early suppression/adaptation and later recovery; and whether MYC/mTORC1 varied with regenerative compartment. Existing GSE225766 niche definitions were not changed to improve agreement. Five-collagen changes were calculated separately for GSE225766 mdx-WT, GSE225766 normal Day5-Day1, GSE161318 Day7-Day5, and GSE223813 Day8-Day4. Magnitudes were interpreted only within study. OXPHOS gene directions and distributions were evaluated independently and compared directionally with the disease anchor.

S10. Human analyses

For GSE288958, MuSCs were identified without diagnosis labels using PAX7-lineage markers and exclusion of other lineages. States were frozen before diagnosis restoration. All 11 donors contributed at least 50 quiescent MuSC nuclei, but only two controls adequately represented early activation. A predefined 211-gene mouse early-activation attenuation module mapped to 197 measurable one-to-one human orthologs. The primary tissue-matched comparison used two quadriceps controls and three DMD donors. Adding three abdominal controls was sensitivity only. The tissue-matched result was negative; the all-control signal was classified as tissue-confounded.

The Heezen spatial dataset contains four control biopsies from three donors and eight dystrophinopathy biopsies from eight donors. HC1 and HC3 share Donor_1 and were averaged after biopsy scoring, yielding 11 donor-level observations. The disease group was analyzed as pooled dystrophinopathy rather than split into underpowered subtype groups. No adequately matched normal injury time course or independent clinical severity endpoint was established from the available inputs.

Whole-biopsy counts were summed over valid in-tissue spots, divided by the total counts across all genes, multiplied by 10^6, and log2(CPM+0.5)-transformed. Each target gene was standardized across biopsies before averaging within its frozen program; repeated-donor biopsy scores were then averaged. Regional scores used all-gene normalization and were averaged across eligible spots before aggregation by donor. Exact two-sided tests enumerate 165 donor-label assignments, using absolute differences in group means. BH correction covers five programs; collagen-gene tests form a separate five-test family, and region tests a joint ten-test family. Approximate Welch intervals are not inversions of permutation tests. Sensitivities include leave-one-donor-out, HC1-only/HC3-only, equal-donor scaling and exclusion of HC2. Whole-biopsy ECM q=0.0303; both regional ECM q=0.0848; excluding HC2 yields q=0.111 with two controls. Age and muscle confounding remain and undermine exchangeability despite the donor correction.

CARD-derived enrichment defined prespecified MuSC-enriched and regeneration-enriched regions. Five programs in two compartments produced ten corrected tests. Positive regional directions were not significant after joint correction (q=0.0848 for both ECM comparisons); enrichment therefore provides exploratory localization only but did not prove MuSC-intrinsic expression because Visium spots are multicellular.

S11. Structured claim audit

Seventy-nine claim records from seven purposively selected dystrophic-muscle publications were compiled before physiological interpretation. Each received one outcome: confirmed unchanged; confirmed but refined; physiologically recontextualized; directionally reinterpreted; spatially/compositionally conditional; or unresolved. Eligibility was claim-specific.

Unresolved was assigned for missing normal activated reference, tissue-mixed controls, one library per genotype-background group, inadequate donor/animal replication, insufficient spatial resolution, or

inadequate state/niche correspondence. Forty-eight claim records were evaluable and formed the denominator for percentages; 31 were retained as unresolved rather than forced into positive or negative outcomes.

Study-panel construction. Seven disease studies were selected purposively from the public datasets used during framework development and validation. Selection required a dystrophinopathy context, accessible molecular data or sufficient published results, interpretable biological metadata, and at least one claim relevant to MuSC state, regeneration, tissue composition, or spatial pathology. The panel was chosen to span modalities and biological contexts; it was not generated by a systematic literature search. Three normal-injury studies were recorded separately as physiological references and were not counted among the seven disease studies.

Claim extraction and freezing. Before examining the physiological-reference classification, each eligible statement was assigned a stable claim identifier and recorded with study, dataset, reported observation, authors' interpretation, original comparison, pathway or process, biological compartment, assay, biological unit and sample structure, source location, URL, and extraction notes. Closely related statements were retained as separate claims only when they referred to distinguishable comparisons, compartments, processes, or evidentiary units. The resulting 79-row master matrix was frozen before outcome assignment.

Standardization. Each record was translated into a testable directional statement while preserving the published observation. Standardization specified what was reported to increase, decrease, persist, fail, or differ; the disease and comparator groups; the relevant cell state, tissue, injury interval, or spatial niche; and whether the evidence was descriptive or replicate-supported.

Reference eligibility and matching. Eligibility was evaluated claim by claim rather than study by study. MuSC-state claims were matched to normal quiescent, early-activation, commitment, cycling, or differentiation

references where available. Temporal claims were matched to normal injury intervals, and spatial claims to validated normal regenerative niches. A claim was not evaluable when the required normal activated state was absent, controls mixed incompatible tissues, biological replication was inadequate, only one library represented a genotype-background group, spatial measurements could not resolve the claimed cellular source, or state/niche correspondence was insufficient.

Disease-effect assessment. The framework did not subtract raw expression values or pathway scores between unrelated platforms. The disease effect was estimated within the source disease dataset using the animal, donor, independent library, or experiment as the statistical unit whenever the design permitted. The selected normal reference was used only to determine the expected physiological direction, timing, state, or niche. Published observations lacking reanalyzable data were interpreted within the evidentiary boundary stated in the source study and were not upgraded to replicate-level inference.

Outcome assignment. Confirmed unchanged indicated that physiological matching did not materially alter the original interpretation. Confirmed but refined indicated that the direction remained supported but its meaning became more specific with respect to magnitude, timing, persistence, resolution, or commitment. Physiologically recontextualized indicated that the observation was substantially compatible with normal regeneration. Directionally reinterpreted was reserved for a change in the biological direction of interpretation; no claim received this outcome. Spatially/compositionally conditional indicated dependence on niche, tissue composition, or cell-state abundance. Unresolved indicated that the available reference or design could not support a defensible interpretation.

Auditability and denominator. Supplementary Table S1 contains one row for each of the 79 frozen claims and preserves the original claim, original comparison, selected normal reference, eligibility decision, matched interpretation, outcome, justification, confidence, source location, and URL. Forty-eight claim records were

evaluable and formed the denominator for percentages among interpretable claims. Thirty-one unresolved records were retained in the full table but excluded from that denominator. Claims from the same publication are not statistically independent; percentages therefore summarize this validation panel only.

S12. Multiple testing and reporting

Benjamini-Hochberg FDR was applied within each frozen test family. Exact donor-level human P values were corrected across five whole-biopsy programs or ten niche-program tests. Significance was interpreted with effect direction, magnitude, member-gene coherence, and replicate stability. Positive mdx-WT denotes higher mdx; interaction effects denote mdx deviation from the corresponding WT transition.

No cell-level P value was treated as biological-replicate inference. One-mouse datasets, one-section time points, and nucleus-level trajectories were explicitly descriptive. Results were downgraded when robust errors, leave-one-out stability, tissue matching, or gene coherence failed.

S13. Interpretation boundary

Physiological matching was used to refine biological interpretation rather than establish causality. Cross-platform raw scores were not treated as directly interchangeable, normal spatial trajectories represented by one section per time point were interpreted descriptively, and enrichment in Visium regions did not establish cellular source or MuSC autonomy. OXPHOS recontextualization was therefore retained as a framework-generated hypothesis rather than a definitive mechanistic conclusion.

S14. Supplementary dataset registry

Dataset	Biological structure	Role
PRJNA771932	**WT n=3; mdx n=4; e23AON-treated mdx n=4**	**MuSC-state analysis, ECM cellular sources, and exploratory**

Dataset	Biological structure	Role
		**communication and treatment analyses**
GSE163856 bulk	n=3-4 per time	Normal acute trajectory
GSE163856 Smart-seq2	2 units/time	Descriptive mapping
GSE268313	3/genotype/state; 24 total	Primary MuSC residual model
GSE268314	1 library/group	Mapping only
GSE225766	WT n=3; D2-mdx n=5	Primary spatial residual model
GSE161318; GSE223813	study-specific normal sections	Independent normal direction
GSE288958	11 human donors	Tissue-matched MuSC stress test
GSE297388	1 library/genotype-background	Descriptive background test
Heezen human spatial	3 control donors (4 biopsies); 8 disease donors; donor-level inference	Limited cross-species association
GSE180771	Four-week and 18-month sedentary cohorts: three WT, three untreated delEx44 and three corrected mice per cohort. Eight-week exercise cohort: two mice per group; descriptive analysis only.	Independent assessment of transcriptional programs following AAV9-CRISPR/Cas9 dystrophin correction.

S15 ECM cellular source

Original singlet counts and annotations were used for 22,417 nuclei from three WT and four mdx mice. Captured transcript contribution was the sum of *Col1a1, Col1a2, Col3a1, Col5a1* and *Col5a2* counts within a lineage divided by their total across all nuclei from that mouse. For cell-type comparisons, an ECM score was the mean log2(CPM+0.5) across these genes, with CPM denominators derived from all genes in the corresponding pseudobulk. Eligibility required ≥20 nuclei in ≥3 mice per genotype. All eligible cell-type tests were BH-adjusted together. Exact tests enumerated genotype assignments while retaining observed group sizes. Diagnostic sensitivities excluded nuclei expressing *Pdgfra, Dcn, Lum, Ptprc or Pecam1* and further required *Pax7 or Myf5* detection. These stringent subsets were descriptive, not a validated ambient-RNA correction.

S16 Human lesion composition

Connective-annotated spots were divided by all valid in-tissue, positive-count spots per biopsy. Corrected whole-biopsy ECM scores were regressed on that fraction within the eight dystrophinopathy samples. Ordinary least squares provided slope and t-based 95% confidence interval (six residual degrees of freedom). Spearman inference enumerated all 8! rank permutations. Leave-one-donor-out slopes assessed direction stability. Annotation fraction was treated as lesion composition, not independent fibrosis.

S17 Regeneration positive controls

Frozen mouse-mapped Hallmark MYOGENESIS, NOTCH_SIGNALING, E2F_TARGETS and G2M_CHECKPOINT genes were applied to log2(TPM+1) expression. Multiple rows mapping to one symbol were collapsed by the mean of log expression. Each gene was standardized across the 24 libraries; scores averaged the measured genes. Models included genotype, state and their interaction, with 16 residual degrees of freedom. Two WT contrasts per program (activated versus freshly isolated MuSCs; myotube versus myoblast) formed an eight-test BH family. The four genotype contrasts per program formed a separate 16-test family. Four omnibus genotype-by-state interaction tests were adjusted together. CAMERA used the count-based edgeR object, the same design/contrasts and inter-gene correlation 0.01, with BH recomputed over the corresponding eight/16-test families. CAMERA tested 196 myogenesis genes, compared with 197 in the TPM score; other set coverage is documented in the tables.

S18 FOXO transcripts and inferred targets

Foxo1/Foxo3 count-based expression contrasts used the existing edgeR quasi-likelihood fit; two genes across four states formed eight BH-adjusted tests. The downloaded CollecTRI mouse network snapshot was restricted to unambiguous activating or inhibiting edges, with duplicates removed. TMM logCPM values were

collapsed by gene symbol using their mean. decoupleR ULM scores required at least five measured signed targets. Genotype-by-state score models and t-based intervals used 16 residual degrees of freedom. Eight tests were adjusted within each specification: all signed targets or targets unique to each FOXO factor. FOXO1/FOXO3 had 182/138 measured signed targets, including 53 shared measured targets; unique-only coverage was 129/85. The raw ULM per-library P values are not disease-comparison P values and must not be substituted for the model contrasts.

S19 Immune satellite communication

LIANA 1.10.0 CellPhoneDB magnitude (`lr_means`) used the saved mouse consensus resource (3,989 pairs). Each mouse was analyzed separately after log1p normalization to 10,000 counts using all genes. Only immune and satellite populations and both cross-population directions were evaluated. Every complex subunit required expression in ≥10% of the relevant population and each population required ≥20 cells. Cell-label permutation P values were not used (`n_perms=None`). Thirty-eight directional pairs were eligible in all seven mice. For each pair, the difference between group mean magnitude scores was assessed against all 35 assignments of three WT/four mdx labels; BH covered all 38 pairs. Ten fixed downsampling seeds selected 20 cells per population per mouse; sensitivity scores used the same fixed eligible interactions without reselecting by expression. Incomplete seven-mouse pair/draws were excluded from sensitivity summaries and retained in a coverage table.

S20 Exploratory exon skipping extension

Four e23AON-treated samples from the same mouse resource were processed with the original basic QC (≥300 detected genes, mitochondrial proportion ≤20%, log10(genes+1)/log10(UMI+1) ≥0.8), followed by sample-wise scDblFinder with fixed seeds. Broad lineage labels were predicted using class-balanced

multinomial logistic regression (C=1, maximum 1,000 iterations) on log1p(CP10k) values for the first 2,000 eligible reference-variable genes, excluding genes in the five original programs and four added controls and mitochondrial/ribosomal genes. Original-mouse labels were predicted out of fold; treated labels used the full reference classifier. Both candidate compartments had to achieve pooled leave-one-mouse-out precision/recall ≥0.8 and ≥20 nuclei with assignment probability ≥0.8 in ≥3 mice of every group. Both passed with three WT/four mdx/four treated mice. Classifier confidence is an operational threshold, not independently calibrated certainty.

For each eligible lineage/mouse, target counts were summed and normalized using all-gene UMI totals. Gene log2(CPM+0.5) values were standardized to the mean/SD of the seven original mice in that lineage, excluding zero-SD genes. Five fixed program scores were modeled as a function of condition (eight residual degrees of freedom), with mdx−WT, treated−mdx and treated−WT contrasts. Each contrast had ten BH-adjusted lineage/program tests. Exact label-permutation sensitivity retained the relevant pairwise group sizes. Reference-based scaling itself was not refitted within permutations; these are fixed-score exploratory tests. Broad-compartment results were not interpreted as state-matched MuSC rescue.

S21 Independent AAV9 CRISPR gene-editing analysis

GSE180771 supplied deposited gene-count matrices and sample-level GEO metadata for bulk quadriceps RNA-seq. The two sedentary cohorts comprised four-week and 18-month mice, each with three WT, three untreated delEx44 and three corrected delEx44 mice. Treatment was delivered at postnatal day 4. The four-week cohort used 8 × 10^{13} vg/kg each of scAAV9-sgRNA and ssAAV9-SpCas9; the 18-month cohort used 2.5 × 10^{14} vg/kg ssAAV9-sgRNA and 5 × 10^{13} vg/kg ssAAV9-SpCas9. Cohorts were analyzed separately because age, dose and vector configuration differed. Six eight-week exercise samples (two/group) were

retained in descriptive score tables only: GEO platform annotations were imbalanced across groups (WT and corrected on NextSeq 550; untreated on NextSeq 500). They were not treated as a third inferential cohort or as longitudinally paired observations.

Before program testing, the analysis specification fixed the original five gene sets and all three contrasts: untreated−WT, corrected−untreated and corrected−WT. Gene definitions were copied from the existing frozen mouse manifest; the ECM set contained *Col1a1, Col1a2, Col3a1, Col5a1 and Col5a2.* Deposited integer counts were checked for duplicate genes, missing samples and negative values. Within each cohort, edgeR filterByExpr used the three-group design, followed by TMM library normalization. LogCPM values used prior.count=0.5 and each retained gene was standardized across all cohort samples, without using group labels for scaling. A program score was the mean of its retained gene z scores. All five ECM genes were retained; full gene coverage and library QC are provided in coverage.csv and qc.csv. Score units are cohort-specific and are neither log fold changes nor Cohen d.

Score models used score ~ group with six residual degrees of freedom in each sedentary cohort, pooled residual variance and t-based 95% confidence intervals. BH correction covered all ten program/cohort tests for each contrast separately. Competitive CAMERA used the same filtered TMM-normalized DGEList, three-group design and contrasts, with its default inter-gene correlation of 0.01; CAMERA P values were separately adjusted across the same ten-test families. Exact two-sided pairwise label permutations enumerated all 20 assignments of three versus three mice, retaining the fixed scores. Because complementary assignments have equal absolute differences, the minimum attainable exact P is 0.10. None of these sensitivity tests can reach $P<0.05$ at this sample size. Model-based significance is therefore reported with this qualification, rather than as permutation-confirmed evidence.
